# Photodynamic Inactivation of Microorganisms Using Semisynthetic Chlorophyll *a* Derivatives as Photosensitizers

**DOI:** 10.3390/molecules27185769

**Published:** 2022-09-06

**Authors:** Marciana Pierina Uliana, Andréia da Cruz Rodrigues, Bruno Andrade Ono, Sebastião Pratavieira, Kleber Thiago de Oliveira, Cristina Kurachi

**Affiliations:** 1 Instituto de Física de São Carlos, Universidade de São Paulo, São Carlos, São Paulo CEP 13560-970, Brazil; 2 Departamento de Química, Universidade Federal de São Carlos, Rodovia Washington Luís, km 235-SP-310, São Carlos, São Paulo CEP 13565-905, Brazil; 3 Universidade Federal da Integração Latino-Americana, Foz do Iguaçu CEP 85866-000, Brazil

**Keywords:** *Spirulina maxima*, chlorophyll *a* derivatives, photosensitizers, semisynthesis, photodynamic inactivation

## Abstract

In this study, we describe the semisynthesis of cost-effective photosensitizers (PSs) derived from chlorophyll a containing different substituents and using previously described methods from the literature. We compared their structures when used in photodynamic inactivation (PDI) against *Staphylococcus aureus*, *Escherichia coli*, and *Candida albicans* under different conditions. The PSs containing carboxylic acids and butyl groups were highly effective against *S. aureus* and *C. albicans* following our PDI protocol. Overall, our results indicate that these nature-inspired PSs are a promising alternative to selectively inactivate microorganisms using PDI.

## 1. Introduction

Photodynamic reactions have been demonstrated to be an efficient alternative for the treatment of cancer [1,2,3,4,5], psoriasis [6], herpes [7], dermatological treatment [8], periodontics [9], canine otitis [10], control of cariogenic bacteria [11,12], and pneumonia [13]. In particular, antimicrobial photodynamic therapy (aPDT) or photodynamic inactivation (PDI) represents an interesting alternative for microbiological control because this technique is minimally toxic, noninvasive, minimizes the use of antibiotics, and due to its action over a broad spectrum of biomolecules, the risk of resistance is unlikely [14,15,16,17,18].

PDI has been used in the treatment of diseases caused by various microorganisms, including Gram-positive and Gram-negative bacteria and fungi [19], such as the inactivation of *Candida albicans*, which causes diseases in patients with low immunity [20,21]. It has also been used in the inactivation of *Aedes aegypti* mosquito larvae, a vector of the dengue, Zika, and chikungunya arboviruses [22,23,24,25,26]. Furthermore, PDI has gained attention in the inactivation of viruses, which have also shown resistance to drugs, such as an alternative to antiviral treatments against human papillomavirus and hepatitis B virus [27]. Recent studies have also shown the use of PDI for the treatment of severe acute respiratory syndrome caused by the new coronavirus (COVID-19) [28]. As PDI can inactivate DNA- or RNA-based viruses, these studies suggest considerable potential for use in virus photoinactivation in the future [29].

In general, photodynamic reactions require the presence of a photosensitizer, which is activated by light at a specific wavelength, allowing the production of reactive oxygen species (ROS). The main ROS are singlet oxygen, superoxide anions, hydroxyl radicals, and hydrogen peroxide. The quantum yields of each of these ROS depend on the PS and the conditions of the medium. After the formation of ROS, they interact with the target cells, causing death [30,31,32,33,34].

The most commonly used photosensitizers are methylene blue [35], porphyrins [36], chlorins [16,37], curcuminoids [38,39], and bacteriochlorins [40]. However, natural product derivatives, such as chlorin-e6 (chl-e6) obtained from chlorophyll a, are relatively low-cost photosensitizers and present many advantages in terms of pharmacokinetics, as they are easily eliminated from the body [11,41].

Chlorophyll a is formally a chlorin derivative, with four nitrogen atoms surrounding a central magnesium atom, along with numerous attached side chains and a hydrocarbon chain. Chlorins are excellent photosensitizers, and several synthetic chlorin analogues, such as m-tetrahydroxyphenylchlorin and mono-L aspartyl chlorin e6, have been used. Some substances, such as porphyrins, chlorins, and bacteriochlorins, stand out for their application in PDI: they present selected photophysical characteristics, allowing structural modifications to promote better solubility and amphiphilicity and improve their properties in several treatments [42]. Natural products are sources of inspiration for the development of several drugs [43]. The natural chlorophyll pigments from the cyanobacterium *Spirulina maxima* are abundant and easy to obtain. According to our protocol, [44] once dried, *S. maxima* is treated with methanol with 5% sulfuric acid, it gives rise to methyl pheophorbide *a*, and, after treatment with different amines or molecular oxygen, generates methyl pheophorbide *a* derivatives, including purpurin-18.

In this study, we propose the diverse semisynthesis of chlorophyll *a* derivatives, using simple reactions to evaluate these photosensitizers against microorganisms. All the chemical modifications performed with chlorophyll a were also aimed at conferring amphiphilicity to the PS, as this strategy has succeeded well in many approaches for PDT or PDI studies [45,46,47,48].

## 2. Results and Discussion

### 2.1. Semisynthesis of Photosensitizers

**Scheme 1:**
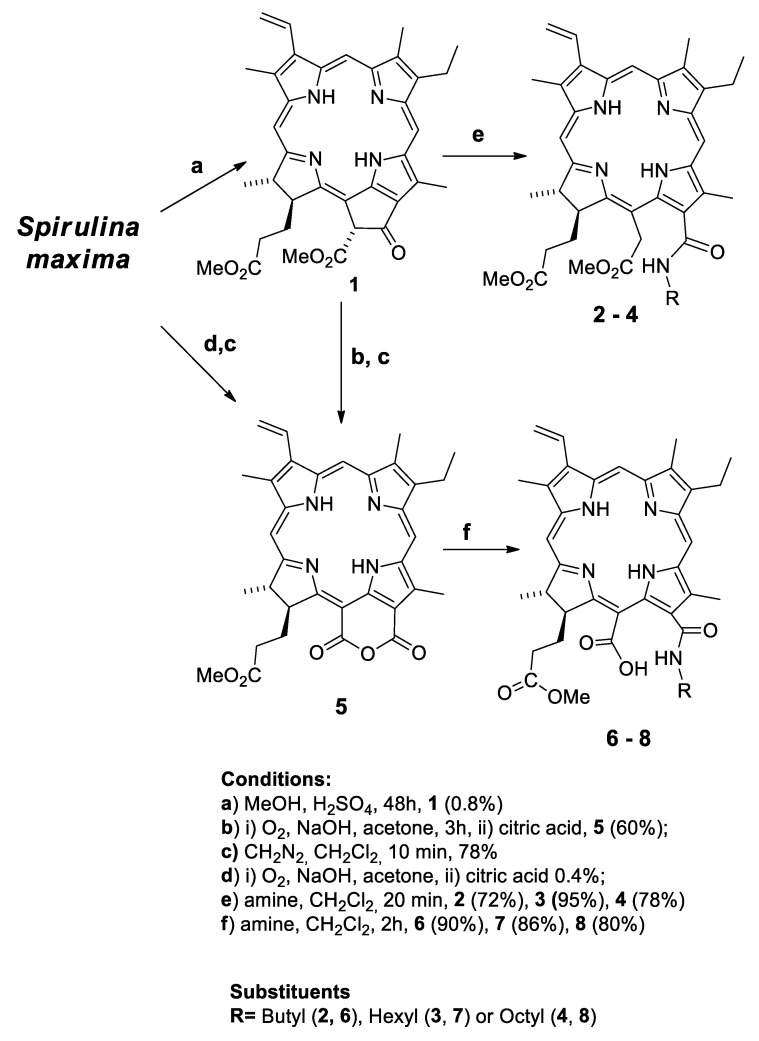
Semisynthesis of chlorophyll *a* derivatives.

All the compounds were prepared using previously described methods from the literature. Initially, methyl-pheophorbide *a* (**1**) was obtained as previously described by our research group [44] (Scheme 1). Subsequently, primary aliphatic amines (butylamine, hexylamine, and octylamine) were reacted with **1** to give compounds **2**–**4**, all with absorption bands around 660 nm [49,50,51].

The purpurin-18 methyl ester (**5**) was also obtained from the oxidation of methyl pheophorbide *a* (**1**) [52,53], which allowed the addition of different aliphatic amines (butylamine, hexylamine, and octylamine) into the anhydride ring, resulting in products **6**–**8**. Compound **5** exhibited a characteristic absorption at 700 nm, which became approximately 660 nm when the anhydride ring was opened by the insertion of the amines (products **6**–**8**) [54,55,56].

**Figure 1:**
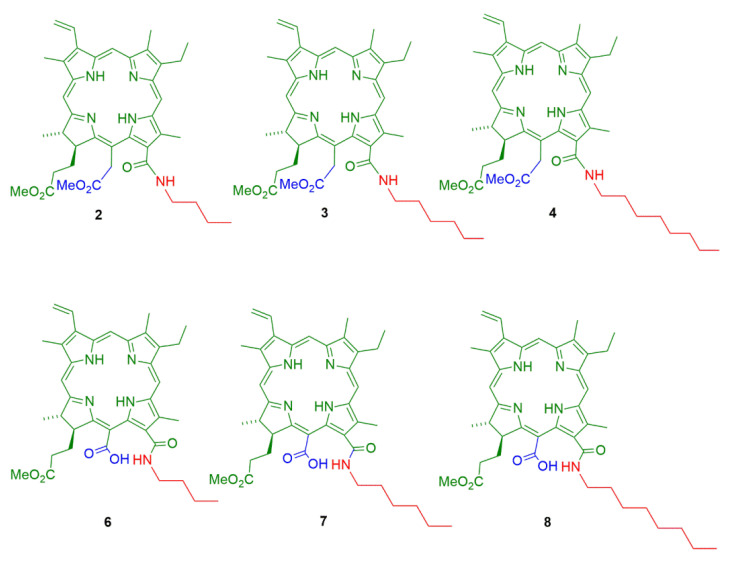
Chlorophyll *a* derivatives **2**–**4** and **6**–**8**.

**Figure 2:**
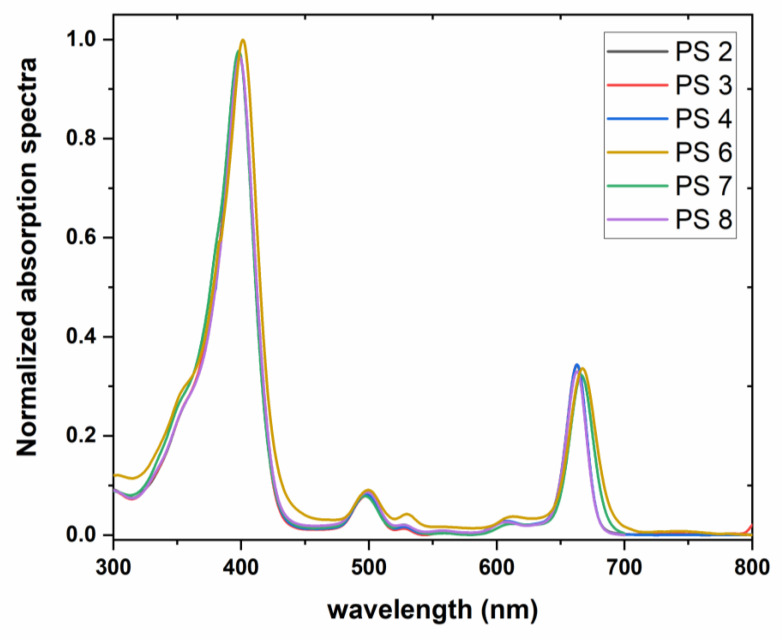
UV-vis absorption spectra of chlorophyll *a* derivatives **2**–**4** and **6**–**8** in ethyl acetate.

The obtained PS **2**–**4** and **6**–**8**, were characterized using NMR, UV-vis, and high-resolution mass spectrometry (HRMS Q-TOFF) and presented absorption bands in the red region. See more details on the characterizations in Supplementary Materials, in which we present our compound band wavelengths and additional literature data (Table S1). Overall, these photosensitizers were semisynthesized at a low cost because we performed small structural modifications in the natural chlorophyll a, having the methyl-pheophorbide *a* (**1**) as a direct and versatile molecular template. With these modifications, we obtained six chlorin derivatives, **2**–**4** and **6**–**8,** all with absorption bands near 660 nm, different substituents, and amphiphilicity, which are desirable for use in PDT (Figure 1 and Figure 2) [57]. The photostability of all compounds was also checked, showing that the photobleaching was not measurable even after 10 min of irradiation (see Supplementary Materials, Figure S18).

### 2.2. Photodynamic Inactivation

**Figure 3:**
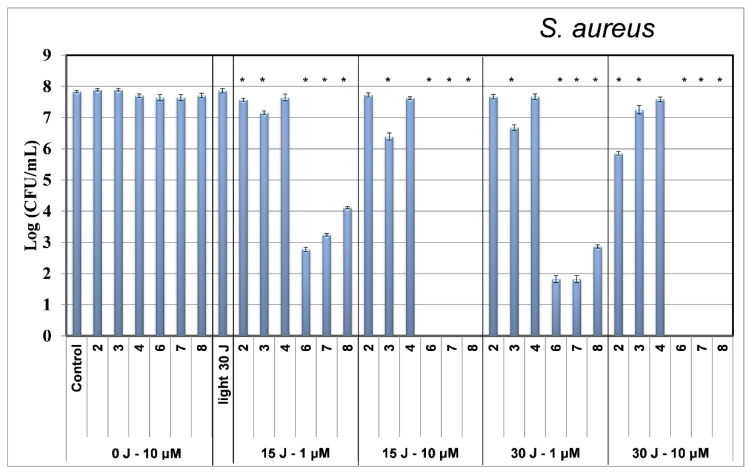
*Staphyloccocus aureus* counts (log CFU/mL) for 1 µM and 10 µM of photosensitizers **2**–**4** and **6**–**8** with the light doses of 0 Jcm^−2^ (control), 15 Jcm^−2^ and 30 Jcm^−2^. Results are presented as mean ± SE. All the experiments were statistically evaluated (Tukey’s test) and are in agreement with significance level of at least *p* < 0.05. Experiments pointed out with (*) represent *p* < 0.05 when compared to the control.

The compounds **2**–**4** and **6**–**8** were evaluated against three microorganisms—*S. aureus*, *C. albicans*, and *E. coli*—for the inactivation of these microorganisms. First, we investigated the dark toxicity of the chlorophyll derivatives. The microorganisms were incubated for 20 min in the dark with the respective photosensitizer (10 μM) and their mortality was evaluated after 24 h. No mortality was observed after 24 h with only the irradiation of the microorganisms without photosensitizers (30 Jcm^−2^). The inactivation study was then performed with each microorganism. All photosensitizers **2**–**4** and **6**–**8** were used in the evaluation of *S. aureus* (Gram-positive bacteria), and different concentrations of photosensitizer (1 μM and 10 μM) and light fluences (15 Jcm^−2^ and 30 Jcm^−2^) were utilized. The results obtained from these initial photoinactivation studies (Figure 3) show that with the increase in the carbon chain (from four to eight C atoms), the photosensitizers presented a decrease in the inactivation levels for the derivatives **2**–**4** and **6**–**8**.

**Figure 4:**
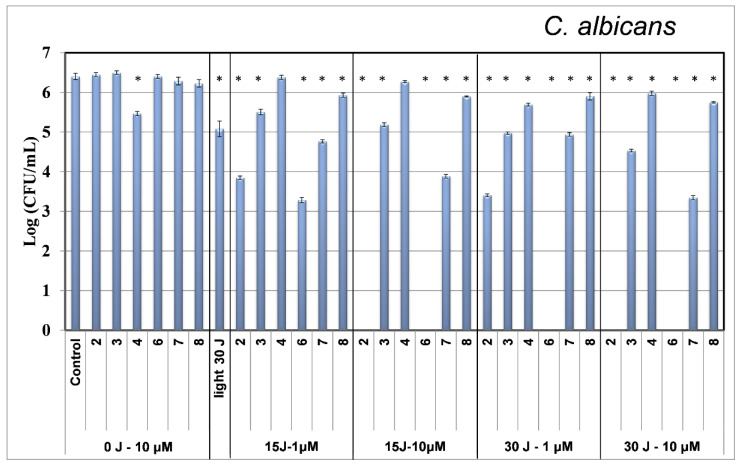
*Candida albicans* counts (log CFU/mL) for 1 µM and 10 µM of photosensitizers **2**–**4** and **6**–**8** with the light doses of 0 Jcm^−2^ (control), 15 Jcm^−2^ and 30 Jcm^−2^. Results are presented as mean ± SE. All the experiments were statistically evaluated (Tukey’s test) and are in agreement with significance level of at least *p* < 0.05. Experiments pointed out with (*) represent *p* < 0.05 when compared to the control.

In addition, we observed that the photoinactivation of both *S. aureus* (Figure 3) and *C. albicans* (Figure 4) was influenced by light fluence and photosensitizer concentration, with increased photoinactivation at high concentrations and fluences.

Evaluating the photoinactivation of *S. aureus* (Figure 3), we observed that at 1 µM and 15 Jcm^−2^, the methyl pheophorbide derivatives **2**–**4** did not present relevant inactivation, whereas the purpurin-18 derivatives **6**–**8** allowed significant inactivation, with a reduction of 3 log using PS **8**, 3.5 log with PS **7,** and 4 log with PS **6**. Maintaining the same concentration of photosensitizer and increasing the dose of light from 15 Jcm^−2^ to 30 Jcm^−2^ resulted in the inactivation of microorganisms by derivatives **2**–**4**; however, the photoinactivation promoted by PS **8** was approximately 4 log, and that by derivatives **6** and **7** was approximately 5 log.

It is possible to observe in Figure 3 that at 10 µM, both light doses (15 Jcm^−2^ and 30 Jcm^−2^) were not very effective in the photoinactivation of *S. aureus* with photosensitizers **2**–**4**. In contrast, purpurin-18 derivatives **6**–**8** completely inhibited the growth of these microorganisms, proving that these derivatives are much more effective than those derived from methyl pheophorbide **2**–**4**.

These results suggest that the purpurin-18 derivatives **6**–**8**, due to the presence of a carboxylic acid group in the molecules may facilitate their microorganism uptake, whereas the derivatives of methyl pheophorbide **2**–**4** have an ester group with lower uptake.

The results presented in Figure 4 for *C. albicans* were similar to those obtained for *S. aureus*, with purpurin-18 derivatives **6**–**8** presenting better results in terms of photoactivation than methyl pheophorbide *a* derivatives **2**–**4**. However, PS **2** with the ester and butyl group also showed promising inactivation of *C. albicans*.

Overall, the results were similar to those for *S. aureus* and the higher the carbon chain present in the photosensitizers, the more hydrophobic the PS is used, and lower microorganism uptake was observed. As a consequence, we observed lower photoinactivation.

Photosensitizers **2**–**4** and **6**–**8** were also tested against *E. coli,* (Gram-negative bacteria), at 500 μM and light dose of 45 Jcm^−2^. No photoinactivation by the photosensitizers **2**–**4** and **6**–**8** was observed, even using high doses of light, which was completely expected as Gram-negative microorganisms are preferentially inactivated by cationic photosensitizers [58,59]. Compared with our previous results using chlorin e6 (**chl-e6**) [44], we observed that *S. aureus* was completely inactivated at **chl-e6** concentrations of 1.6 μM and 16 μM when using a light dose of 30 Jcm^−2^, which is very similar to the results obtained with derivatives **6** and **7**. However, considering *C. albicans* when compared with **chl-e6**, we found total inactivation at concentrations equivalent to 33 μM (20 μg mL^−1^) and 50 μM (30 μg mL^−1^) using a light dose of 30 Jcm^−2^. We obtained the best results with derivative **6**, which completely inactivated *C. albicans* at a concentration of 10 μM (15 Jcm^−2^) and at concentrations of 1 μM and 10 μM with 30 Jcm^−2^. Chlorin e6 and **2**, **3**, **4**, **6**, **7**, and **8** did not show efficacy against *E. coli* [44].

Comparing among photosensitizers, when methylene blue was evaluated against the microorganism *S. aureus* at a concentration of 50 μM and a light dose of 9 J at 660 nm [60], a reduction of approximately 1.5 log CFU was obtained, whereas the photosensitizers **6**, **7**, and **8** used in this study completely inactivated the *S. aureus* microorganism at a concentration of 10 μM and a light dose of 15 Jcm^−2^, demonstrating it was more effective using these semisynthetic PS. When using 50 μM and a light dose of 9 J to perform photodynamic inactivation with methylene blue for *C. albicans*, a reduction of approximately 1 log CFU was obtained, whereas in this study, PS **7** at a concentration of 10 μM and a light dose of 15 Jcm^−2^ allowed a reduction of approximately 2 log CFU. Using the PS **3**, we obtained a reduction of approximately 1 log CFU, and PS **2** and **6** completely inactivated the microorganism.

Indocyanine green (ICG) [61], was also used as a photosensitizer for the photoinactivation of *S. aureus*. At a concentration of 25 μg mL^−1^ (32 μM) ICG and exposure to 411 Jcm^−2^ with near-infrared (NIR) light (808 nm laser), a significant reduction in the viable count was achieved (5.56 log10); complete inactivation of the microorganism occurred at a concentration of 200 μg mL^−1^ (258 μM) and 411 Jcm^−2^. In another study using ICG [62], PDI against *S. aureus* with an ICG concentration of 8 μg mL^−1^ (10 μM) at an energy dose of 84 Jcm^−2^ resulted in 100% inactivation of this microorganism. In the current study, PS **6**, **7**, and **8** also showed complete inactivation of *S. aureus* at a concentration of 10 μM using 15 Jcm^−2^ and 30 Jcm^−2^ (660 nm).

In an in vitro study, the inactivation of *C. albicans* was evaluated with ICG at 1 μg mL^−1^ using a light dose of 228 J/cm^−2^ (810 nm); a satisfactory result showing a reduction of 1.2 log was obtained, similar to the results with nystatin. Compared to the control, the elimination of *C. albicans* increased by 92% when treated with ICG (1 mg/mL) with infrared (IR) laser irradiation (810 nm, 55 J/cm^−2^).

## 3. Materials and Methods

Nuclear magnetic resonance (NMR) analyses were performed on a Bruker Avance 400 spectrometer at 400.15 MHz (^1^H) and 100.13 MHz (^13^C). Tetramethylsilane was used as an internal reference.

High-resolution mass spectrometry (HRMS) was performed using ESI-TOF (Waters Xevo G2-S Qtof). Ultraviolet-visible spectrophotometry analyses were performed using a Lambda 25 spectrometer (PerkinElmer, Waltham, MA, USA).

*Spirulina maxima* powder was purchased from Pharma Nostra (Rio de Janeiro, Brazil), and other reagents were purchased from Sigma-Aldrich (St. Louis, MO, USA).

### 3.1. Semisynthesis of Photosensitizers

*Isolation of methyl pheophorbide a* (***1***) *from Spirulina maxima* (*Mepheo* a): 300 g of the *Spirulina maxima* was treated with a 1.5 L of the 5% methanolic solution of H_2_SO_4_ for 24 h at room temperature. This mixture was filtered and washed with methanol (900 mL) and ethyl acetate (900 mL), and the organic phases were evaporated under reduced pressure. After that, 150 g of crushed ice was added to the crude residue. The residue was neutralized with solid NaHCO_3_ and placed on a silica gel plug. The chlorophyll derivatives were retained on the plug, and the residual proteins and peptides (pale yellow in color) were eluted with water. The chlorophyll derivatives were then eluted with ethyl acetate (900 mL) and washed with water (3 × 400 mL). The organic phase was separated, dried over Na_2_SO_4_, and the solvent evaporated under reduced pressure. The methyl pheophorbide *a* was purified by silica gel flash chromatography using as eluent toluene:ethyl acetate (9:1), yielding the methyl pheophorbide *a* (**1**) (2.4 g, 3.9 mmol, 0.8% yield from natural dried *Spirulina maxima*) [44,63,64].

UV-vis (CH_2_Cl_2_): λmax (nm): 666, 609, 534, 505, 409 [65].

^1^H-NMR (CDCl_3_, 400 MHz) δ: 9.51 (s, 1H, H-10); 9.37 (s, 1H, H-5); 8.56 (s, 1H, H-20); 7.97 (dd, 1H, *J* = 11.6, *J* = 17.8 Hz, H-3^1^); 6.25 (s, 1H, H-13^2^); 6.24 (dd, 1H, *J =* 1.5 and *J =* 17.8 Hz, H-3^2B^); 6.16 (dd, 1H, *J* = 1.5 and *J* = 17.8 Hz, H-3^2a^); 4.48–4.43 (m, 1H, H-18^1^); 4.21–4.19 (m, 1H, H-17); 3.88 (s, 3H, H-13^4^); 3.68 (s, 3H, H-12^1^); 3.60 (q, 2H, *J* = 7.7 Hz, H-8^1^); 3.57 (s, 3H, H-17^4^); 3.40 (s, 3H, H-2^1^); 3.22 (s, 3H, H-7^1^); 2.68–2.48 (m, 2H, H-17^1^); 2.36–2.20 (m, 2H, H-17^2^); 1.81 (d, 3H, *J* = 7.3 Hz, H-18^2^); 1.69 (t, 3H, *J* = 7.6 Hz, H-8^2^); 0.54 (br. s, 1H, H-21); −1.63 (br. s, 1H, H-23).

^13^C-NMR (CDCl_3_, 100 MHz) δ: 192.1, 174.7, 172.5, 169.4, 162.9, 155.9, 151.1, 150.1, 145.3, 142.3, 137.9, 136.8, 136.4, 136.3, 132.0, 129.1, 129.0, 127.9, 122.9, 104.6, 104.5, 97.5, 93.4, 65.9, 65.1, 63.3, 52.2, 51.1, 50.3, 30.8, 30.0, 23.2, 19.4, 17.4, 12.1, 11.3

*13-(Butylcarbamoyl)-chlorin e6 15,17-dimethyl ester* (***2***). To a solution of 30 mg (0.049 mmol) of **1** in 3 mL of dry tetrahydrofuran, 0.5 mL of butylamine was added and the reaction was stirred for 20 min at room temperature. After that, the solvent was evaporated, the mixture was diluted with dichloromethane and washed with HCl solution (1%). The resulting solution was dried over anhydrous sodium sulfate, and the solvent evaporated under reduced pressure. Compound 2 was isolated by chromatography over silica gel using toluene:ethyl acetate (9:1) as eluent (22 mg, 72% yield) [50,51].

UV-vis (CH_2_Cl_2_) λmax (nm): 662, 607, 528, 498, 399 [66].

^1^H-NMR (CDCl_3_, 400 MHz) δ: 9.69 (s, 1H); 9.64 (s, 1H); 8.80 (s, 1H); 8.08 (dd, 1H, *J* = 17.8 and 11.6 Hz); 6.36–6.40 (m, 1H, CONH); 6.33 (dd, 1H, *J* = 16.7, 1.4 Hz); 6.15 (dd, 1H, *J* = 11.6, 1.4 Hz); 5.54 and 5.26 (d, 1H each, *J* = 18.9 Hz); 4.46 (q, 1H, *J* = 7.2 Hz); 4.35 (d, 1H, *J* = 9.5 Hz); 3.91–3.76 (m, 4H); 3.81 (s, 3H); 3.60 (s, 3H); 3.56 (s, 3H); 3.49 (s, 3H); 3.32 (s, 3H); 2.56–2.09 (m, 4H); 1.82–1.69 (m, 10H); 1.06 (t, 3H, *J* = 7.3 Hz); −1.61 (br.s, NH); −1.83 (br.s, NH).

HRMS (ESI): *m*/*z* calculated [M + H]^+^ for C_40_H_50_O_5_N_5_^+^ = 680.38065; found 680.38379.

*13-(Hexylcarbamoyl)chlorin e6 15,17-dimethyl ester* (***3***). To a solution of 40 mg (0.066 mmol) of **1** in 3 mL of dry tetrahydrofuran, 0.5 mL of hexylamine was added and the reaction was stirred for 20 min at room temperature. After that, the solvent was evaporated, the mixture was diluted with dichloromethane and washed with HCl solution (1%). The resulting solution was dried over anhydrous sodium sulfate, and the solvent evaporated under reduced pressure. Compound 3 was isolated by chromatography over silica gel using toluene:ethyl acetate (9:1) as eluent (45 mg, 95% yield) [50,51].

UV-vis (CH_2_Cl_2_): λmax (nm): 663, 605, 527, 497, 398 [66].

^1^H-NMR (CDCl_3_, 400 MHz) δ: 9.70 (s, 1H), 9.64 (s, 1H), 8.80 (s, 1H), 8.10 (dd,1H, *J* = 11.5 and 17.8 Hz), 6.36–6.39 (m, CONH), 6.35 (dd, 1H, *J* = 1.4 and 17.8 Hz), 6.13 (dd, 1H, *J* = 1.4 and 11.5 Hz), 5.52 (d, 1H, *J* = 18.9 Hz), 5.25 (d, 1H, *J* = 19.0 Hz), 4.46 (q, 1H, *J* = 7.3 Hz), 4.35 (d, 1H, *J* = 11.2 Hz), 3.90–3.68 (m, 4H), 3.80 (s, 3H), 3.60 (s, 3H), 3.57 (s, 3H), 3.49 (s, 3H), 3.32 (s, 3H), 2.56–2.07 (m, 4H), 1.83–1.37 (m, 16H), 0.94 (t, 1H, *J* = 7.2 Hz), −1.63 (NH), −1.83 (NH).

HRMS (ESI): m/z calculated [M + H]^+^ for C_42_H_54_O_5_N_5_^+^ = 708.41195; found 708.41516.

*13-(Octylcarbamoyl)chlorin e6 15,17-dimethyl ester* (***4***). To a solution of 30 mg (0.049 mmol) of **1** in 3 mL of dry tetrahydrofuran, 0.5 mL of octylamine was added and the reaction was stirred for 20 min at room temperature. After that, the solvent was evaporated, the mixture was diluted with dichloromethane and washed with HCl solution (1%). The resulting solution was dried over anhydrous sodium sulfate, and the solvent evaporated under reduced pressure. Compound 4 was isolated by chromatography over silica gel using toluene:ethyl acetate (9:1) as eluent (28 mg, 78% yield) [51].

UV-vis (CH_2_Cl_2_): λmax (nm): 663, 609, 527, 497, 399 [66].

^1^H-NMR (CDCl_3_, 400 MHz) δ: 9.70 (s, 1H); 9.64 (s, 1H); 8.80 (s, 1H); 8.09 (dd, 1H, *J* = 17.8, 11.5 Hz); 6.36–6.39 (m, 1H, CONH); 6.36 (dd, 1H, *J* = 18.2, 1.4 Hz); 6.14 (dd, 1H, *J* = 11.5, 1.4 Hz), 5.54 and 2.25 (d, 1H each, *J* = 18.9 Hz); 4.47 (q, 1H, *J* = 7.1 Hz); 4.36 (d. 1H, *J* = 7.6 Hz); 3.90–3.77 (m, 4H); 3.80 (s, 3H); 3.60 (s, 3H); 3.57 (s, 3H); 3.49 (s, 3H); 3.32 (s, 3H); 2.52–2.09 (m, 4H); 1.83–1.69 (m, 10 H); 1.46–1.33 (m, 8H); 0.90 (t, 3H, *J* = 7.1 Hz); −1.62 (br, s, NH); −1.83 (br, s, NH).

HRMS (ESI): *m*/*z* calculated [M + H]^+^ for C_44_H_58_O_5_N_5_^+^ = 736.44325; found 736.44147.

*Purpurin-18 Methyl Ester* (***5***) Method 1: Methyl pheophorbide *a* **1** (151 mg, 0.25 mmol) was dissolved in 500 mL of diethyl ether and 5 mL of pyridine. After, a potassium hydroxide solution in 1-propanol (2 g of KOH was dissolved in 10 mL of 1-propanol) was added into the first solution and oxygen was bubbled into the resulting reaction mixture for 1 h. The reaction mixture was extracted with water (500 mL). The aqueous layer was collected and the pH adjusted to 2–4 using cold H_2_SO_4_ solution (25%). The aqueous layer was extracted with CH_2_Cl_2_ and the solvent evaporated to give a purple residue. The product was purified by chromatography over silica gel using hexane:ethyl acetate 3:1 as eluent. After that, the carboxylic acid precursor was obtained in 55% yield (0.078 g, 0.119 mmol). This product was further reacted with a diazomethane solution in dichloromethane to produce purpurin-18 methyl ester (**5**) for 10 min at 0 ºC. The residue was crystallized with dichloromethane/hexane, thus obtaining **5** in 60% yield (63.0 mg, 0.109 mmol) as purple red crystals [51]. Method 2: Pigments were extracted twice from *S. maxima* dried powder (10 g) with acetone (4 × 100 mL) under magnetic stirring at 60 °C (4 × 30 min). The dark green extract was filtered off and the filtrate (ca. 400 mL) was reduced to 200 mL by partial evaporation under reduced pressure. NaOH (40 mL, 6 M) was added to the previous extract (200 mL); the mixture was vigorously stirred and oxygen was bubbled during 3 h. The solution was then acidified with concentrated HCl. The oxidized extract was evaporated to dryness. Carotenoids and part of xanthophylls were removed by extraction with petroleum ether (2 × 100 mL). The resulting residue was purified by flash chromatography (eluent CH_2_Cl_2_:MeOH 8:2) obtaining 40.0 mg (0.070 mmol, 0.4% yield) of the purpurin-18 carboxylic acid. The product was esterified with diazomethane obtaining 32.0 mg (0.055 mmol, 78% yield) of the 5 [53,67].

UV-vis (CH_2_Cl_2_): λmax (nm): 699, 642, 546, 508, 478, 410.

^1^-H NMR (400 MHz, CDCl_3_, ppm): 9.57 (s, 1H, H-10); 9.36 (s, 1H, H-5); 8.56 (s, 1H, H-20); 7.89 (dd, 1H, *J* = 17.8, 11.6 Hz, H-3^1^); 6.27 (dd, 1H, *J =* 17.8 and *J* = 9.4, 2.5 Hz, H-3^2B^); 6.20 (dd, 1H, *J =* 11.6 and *J* = 9.4, 2.5 Hz, H-3^2a^); 4.39 (q, 1H, *J* = 7.3 Hz, H-18^1^); 3.77 (s, 3H, H-12^1^); 3.64 (q, 2H, *J* = 7.9 Hz, H-8^1^); 3.60 (s, 3H, H-17^4^); 3.35 (s, 3H, H-2^1^); 3.15 (s, 3H, H-7^1^); 2.78–2.70 (m, 2H H-17^1^); 2.49–2.43 (m, 2H, H-17^2^); 1.74 (d, 3H, *J =* 7.3 Hz, H-18^2^); 1.65 (t, 3H, *J* = 7.6 Hz, H-8^2^); 0.21 (br s, 1H, H-21); −0.08 (br s, 1H, H-23).

^13^C-NMR (CDCl_3_, 100 MHz) δ: 178.6, 173.5, 169.8, 165.4, 163.4, 158.4, 154.2, 146.9, 145.1, 141.9, 140.0, 137.9, 135.5, 133.5, 132.8, 132.8, 131.4, 128.0, 125.6, 107.4, 102.5, 97.9, 94.5, 55.5, 51.7, 49.6, 32.4, 31.4, 20.4, 19.6, 16.9, 12.9, 12.1, 11.2

*Chlorin-p, 6-N-Butylamide-7-methyl Ester* (***6***). To a solution of **5** (40.0 mg, 0.069 mmol) in dichloromethane (5 mL), butylamine *(*0.5 mL) was added and the reaction mixture was stirred at room temperature in the dark under nitrogen for 2 h. After that, the UV-vis analysis and TLC showed the absence of starting material. The product was purified by crystallization with dichloromethane-hexane to give the product **6** (48.0 mg, 0.070 mmol, 90% yield) [54,55,56].

UV-vis (CH_2_Cl_2_): λmax (nm): 662, 606, 526, 498, 398.

^1^H-NMR (CDCl_3_, 400 MHz) δ: 9.67(s, 1H), 9.62 (s, 1H), 8.74 (s, 1H), 8.12 (dd, *J* = 18, 11 Hz 1H), 6.96–7.05 (m, NH), 6.37 (d, *J* = 18 Hz, 1H), 6.15 (d, *J* = 11 Hz, 1H), 5.06 (d, *J* = 7.7 Hz,1H), 4.33–4.37 (m, 1H) 3.74–3.78 (m, 4H), 3.57 (s, 3H), 3.46 (s, 3H), 3.31 (s, 3H), 3.23 (s, 3H), 2.49–2.27 (m, 2H), 1.70 (d, 3H, *J* = 7.2Hz), 1.68 (t, 3H, *J* = 7.5Hz), 0.79–1.10 (m, 4H), 0.95 (t, 3H, *J* = 7.2Hz), 0.63(t, 3H, *J* = 7.2Hz),−1.66 (s, NH) and −1.94 (s, NH).

HRMS (ESI): *m*/*z* calculated [M + H]^+^ for C_38_H_46_O_5_N_5_^+^ = 652.34935; found 652.35046.

*Chlorin-p, 6-N-hexylamide-7-methyl Ester* (***7***). To a solution of **5** (40 mg, 0.069 mmol) in dichloromethane (0.3 mL), hexylamine *(*0.2 mL) was added and the reaction mixture was stirred at room temperature in the dark under nitrogen for 2 h. After that, UV-vis analysis and TLC showed the absence of starting material. The product was purified by crystallization with dichloromethane-hexane to give the product **7** (42.0 mg, 0.062 mmol, 86% yield) [54,55,56].

UV-vis (CH_2_Cl_2_): λmax (nm): 666, 608, 528, 497, 398.

^1^H-NMR (CDCl_3_, 400 MHz) δ: 9.66 (s, 1H), 9.55 (s, 1H), 8.70 (s, 1H), 7.98 (dd, 1H, *J* = 11.5 and 17.6 Hz), 6.33–6.37 (m, NH), 6.28 (d, 1H, *J* = 16.8Hz), 6.10 (d, 1H, *J* = 12.8Hz), 4.93 (d, 1H, *J* = 7.7Hz), 4.40 (q, 1H, *J* = 7.0Hz), 4.13 (s, 3H), 3.82–3.60 (m, 4H), 3.56 (s, 3H), 3.40 (s, 3H), 3.22 (s, 3H), 2.70–2.12 (m, 4H), 1.77 (d, 3H, *J* = 7.2Hz), 1.64 (t, 3H, *J* = 7.5Hz), 0.92–0.77 (m, 9H), 0.67 (t, 3H, *J* = 7.2Hz), −1.32 (s, NH), −1.54 (s, NH).

HRMS (ESI): *m*/*z* calculated [M + H]^+^ for C_40_H_50_O_5_N_5_^+^ = 680.38065; found 680.38251.

*Chlorin-p, 6-N-octylamide-7-methyl Ester (**8**).* To a solution of **5** (22.0 mg, 0.038 mmol) in dichloromethane (1.0 mL), octylamine (0.2 mL) was added. The reaction mixture was stirred at room temperature in the dark under nitrogen for 2 h. After that, UV-vis analysis and TLC showed the absence of starting material. The product was purified by crystallization with dichloromethane-hexane to give the product **8** (21.0 mg, 0.030 mmol, 80% yield) [54,55,56].

UV-vis (CH_2_Cl_2_): λmax (nm): 663, 605, 528, 499, 399.

^1^H-NMR (CD_3_)_2_CO, 400 MHz) δ: 9.71 (s, 1H), 9.41 (s, 1H), 9.04 (s, 1H), 8.17 (dd, 1H), 7.78–7.82 (m, NH), 6.37 (d, 1H), 6.15 (d, 1H), 5.16 (d, 1H), 4.54 (q, 1H), 3.67 (s, 3H), 3.60–3.64 (m, 4H), 3.48 (s, 3H), 3.40 (s, 3H), 3.25 (s, 3H), 2.70–2.12 (m, 6H), 1.78 (d, 3H), 1.63 (t, 3H, *J* = 7.5Hz), 0.92–0.77 (m, 9H), 0.85 (t, 3H, *J* = 7.2Hz), −1.46 (s, NH), −1.82 (s, NH).

HRMS (ESI): m/z calculated [M + H]^+^ for C_42_H_54_O_5_N_5_^+^ = 708.41195; found 708.41364.

### 3.2. Photodynamic Inactivation

*Staphylococcus aureus* (American Type Culture Collection, ATCC 25923) and *Escherichia coli* (ATCC 25922) were grown in brain and heart infusion media. *Candida albicans* (ATCC 10231) was grown in Sabouraud dextrose broth. For experimental purposes, the microorganism concentration was adjusted to 10^7^–10^8^ cells/mL in sterile distilled water, and 500 µL of each microorganism culture was added to 24-well plates with photosensitizers **2**–**4** and **6**–**8**. Solutions were prepared by diluting the photosensitizer powder (1 mg for *S. aureus* and *C. albicans* and 2 mg for *E. coli*) in 100 μL of dimethyl sulfoxide (DMSO) (to dissolve the PS) and 900 μL of sterile water; the initial concentration of DMSO in the stock solutions was 10%. After dilution of these stock solutions to final concentrations of 1 µM and 10 µM for *S. aureus* and *C. albicans*, respectively, and of 500 μM for *E. coli,* the final concentration of DMSO was less than 2% in all the solutions. After preparing the solutions, they were protected from light. The 24-well plates were kept in the dark at 37 °C for 20 min.

A homemade LED-based device with emission centered at 660 nm was used to irradiate the culture plates. The 24-well plates were irradiated at 30 mWcm^−2^ using this device for 8, 16, and 25 min, resulting in fluences of 15, 30, and 45 Jcm^−2^, respectively, which were used in the PDI against microorganisms. The fluence levels used were 15 Jcm^−2^ and 30 Jcm^−2^ for *S. aureus* and *C. albicans*, and 45 Jcm^−2^ for *E. coli*. After irradiation, 10-fold serial dilutions were performed and cells were cultured in agar plates. The colony-forming units (CFUs) were determined 24 h after initiation of the experimental procedure. All experiments were performed in triplicate.

In the control group (no treatment), 24-well plates were maintained at room temperature for 32 min. Using the same incubation time, the dark toxicity of the photosensitizers was evaluated in 24-well plates covered with aluminum foil to avoid light exposure. Phototoxicity was determined by irradiation at 30 Jcm^−2^ for *S. aureus* and *C. albicans*, and 45 Jcm^−2^ for *E. coli*.

Survival fractions (SFs) were expressed as ratios of CFUs of treated groups to the control group. The SF at 0 J/cm^2^ provides a measure of the dark toxicity of chlorins.

### 3.3. Statistical Analysis

All the results reported in Figure 3 and Figure 4 were statistically analyzed using the RStudio software (R version 4.1.1 (10 August 2021), R Core Team (2021), R: A language and environment for statistical computing; R Foundation for Statistical Computing, Vienna, Austria (https://www.R-project.org, accessed on 10 August 2021)) using a significance level of at least *p* < 0.05 and a confidence level of approximately 95%. The data were analyzed and approved by the normality test. Comparisons between the experimental groups were verified by Tukey’s test.

## 4. Conclusions

The photosensitizers **2**–**4** and **6**–**7** derived from chlorophyll a were successfully semisynthesized. The characterization of the compounds is in accordance with data described in the literature and the main photophysical data are compiled and organized in Table S1 (SI). Overall, methyl pheophorbide *a* or purpurin-18 derivatives with different side chains (from the butyl to octyl groups) were prepared and studied. Subsequently, we investigated the photoinactivation of *S. aureus* and observed that the methyl pheophorbide derivatives **2**–**4** did not show great inactivation, whereas the purpurin-18 derivatives **6**–**8** allowed significant PDT inactivation. For *C. albicans*, the purpurin-18 derivative with the butyl group showed relevant inactivation, and the methyl pheophorbide with the butyl group also exhibited PDI. Photosensitizers **2**–**4** and **6**–**8** were also tested against the Gram-negative bacterium *E. coli*; however, no significant photoinactivation was observed. In general, the higher the carbon chain present in the photosensitizers, the more hydrophobic the compounds, with consequently lower photoinactivation efficacy. These results suggest that the use of chlorin derivatives with lower hydrophobic properties can be more effective for the photoinactivation of such microorganisms as *S. aureus* and *C. albicans*.

## Data Availability

Not applicable.

## Supplementary Materials

The following supporting information can be downloaded at: https://www.mdpi.com/1420-3049/27/18/5769/s1, Figure S1: ^1^H-NMR (CDCl_3_) spectrum of methyl pheophorbide *a* (1); Figure S2: ^13^C-NMR (CDCl_3_) spectrum of methyl pheophorbide *a* (1); Figure S3: ^1^H-NMR (CDCl_3_) spectrum of 13-(Butylcarbamoyl)chlorin *e*6 15,17-dimethyl ester (2); Figure S4: HRMS (ESI) of 13-(Butylcarbamoyl)chlorin *e*6 15,17-dimethyl ester (2); Figure S5: ^1^H-NMR (CDCl_3_) spectrum of 13-(Hexylcarbamoyl)chlorin *e*6 15,17-dimethyl ester (3); Figure S6: HRMS (ESI) of 13-(Hexylcarbamoyl)chlorin *e*6 15,17-dimethyl ester (3); Figure S7: ^1^H-NMR (CDCl_3_) spectrum of 13-(Octylcarbamoyl)chlorin *e*6 15,17-dimethyl ester (4); Figure S8: HRMS (ESI) of 13-(Octylcarbamoyl)chlorin *e*6 15,17-dimethyl ester (4); Figure S9: ^1^H-NMR (CDCl_3_) spectrum of Purpurin-18 Methyl Ester (5); Figure S10: ^13^C-NMR (CDCl_3_) spectrum of Purpurin-18 Methyl Ester (5); Figure S11: ^1^H-NMR (CDCl_3_) spectrum of Chlorin-p, 6-N-Butylamide-7-methyl Ester (6); Figure S12: HRMS (ESI) of Chlorin-p, 6-N-Butylamide-7-methyl Ester (6); Figure S13: ^1^H-NMR (CDCl_3_) spectrum of Chlorin-p, 6-N-hexylamide-7-methyl Ester (7); Figure S14: HRMS (ESI) of Chlorin-p, 6-N-hexylamide-7-methyl Ester (7); Figure S15: ^1^H-NMR (CDCl_3_) spectrum of Chlorin-p, 6-N-octylamide-7-methyl Ester (8); Figure S16: HRMS (ESI) of Chlorin-p, 6-N-octylamide-7-methyl Ester (8); Figure S17: Homemade engineered Biotable model for PDI studies (660 nm). Figure S18: Photodegradation experiment at 660 nm at 63.7 mWcm-2 using ethyl acetate as solvent. Table S1: Wavelengths data and Quantum yield of Singlet Oxygen ^1^O_2_ data of the literature for some of the compounds.
