# Supplementary material for: Photodynamic Inactivation of Microorganisms Using Semisynthetic Chlorophyll a Derivatives as Photosensitizers
Source: Molecules. 2022 Sep 6;27(18):5769. doi: 10.3390/molecules27185769 (PMC9653790; doi:10.3390/molecules27185769)
Supplement: Supplementary file 1 [file molecules-27-05769-s001.zip › molecules-1836012-supplementary.pdf]

Supporting Information

Photodynamic Inactivation of Microorganisms Using Semi-Synthetic Chlorophyll a Derivatives as Photosensitizers

Marciana Pierina Uliana[1,2,3*], Andréia da Cruz Rodrigues[3], Bruno Andrade Ono[1], Sebastião Pratavieira[1], Kleber Thiago de Oliveira[2] and Cristina Kurachi[1]

1 Instituto de Física de São Carlos, Universidade de São Paulo, CEP 13560-970, São Carlos, SP, Brazil

2 Departamento de Química, Universidade Federal de São Carlos, Rodovia Washington Luís, km 235 - SP-310, 13565-905, São Carlos - SP – Brazil;

3 Universidade Federal da Integração Latino-Americana, CEP 85866-000, Caixa Postal 2044, Foz do Iguaçu, PR, Brazil; marciana.machado@unila.edu.br (M. P. U)

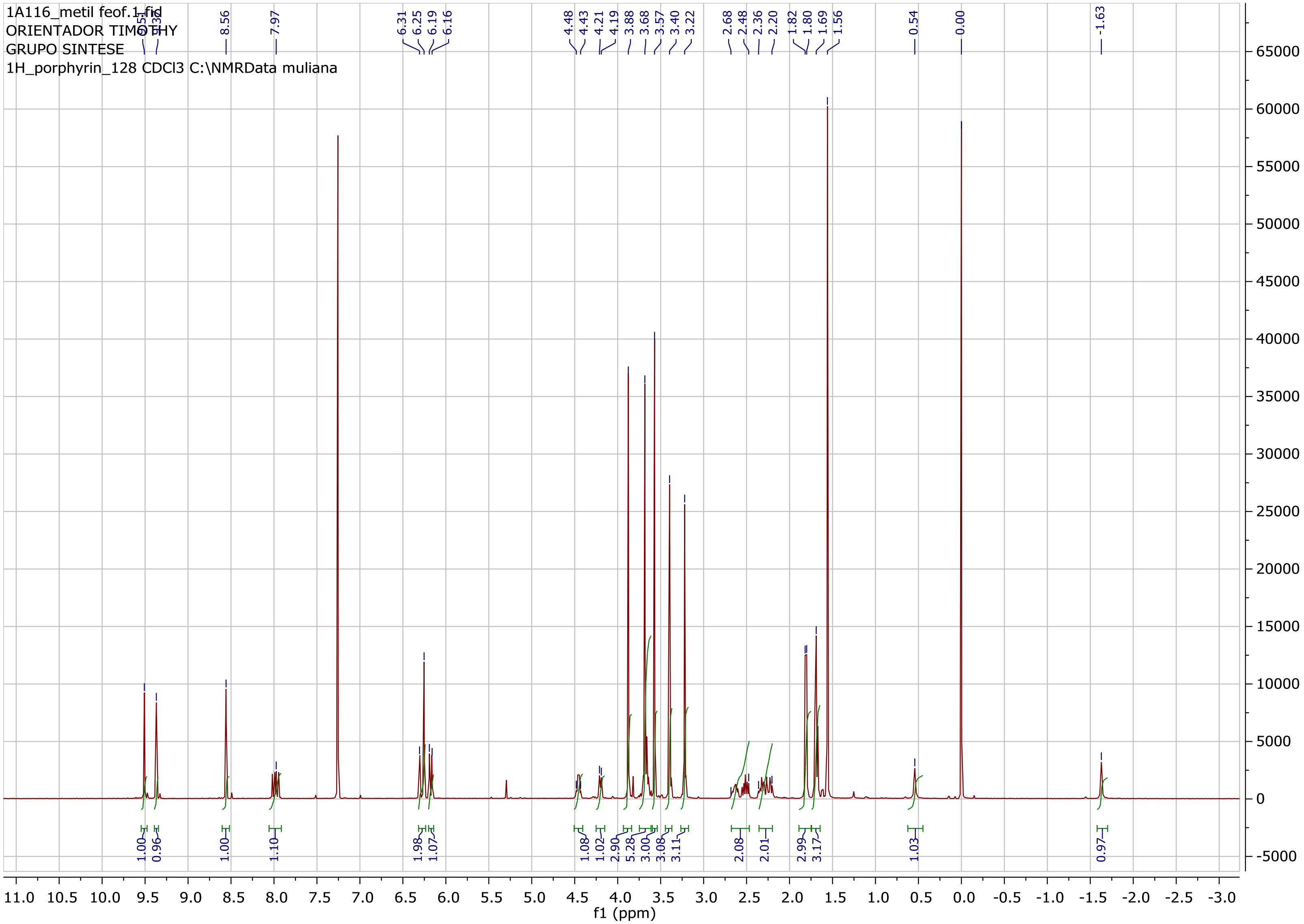

Figure S1: ^{1}H-NMR ($CDCl_3$) spectrum of methyl pheophorbide-*a* (**1**)

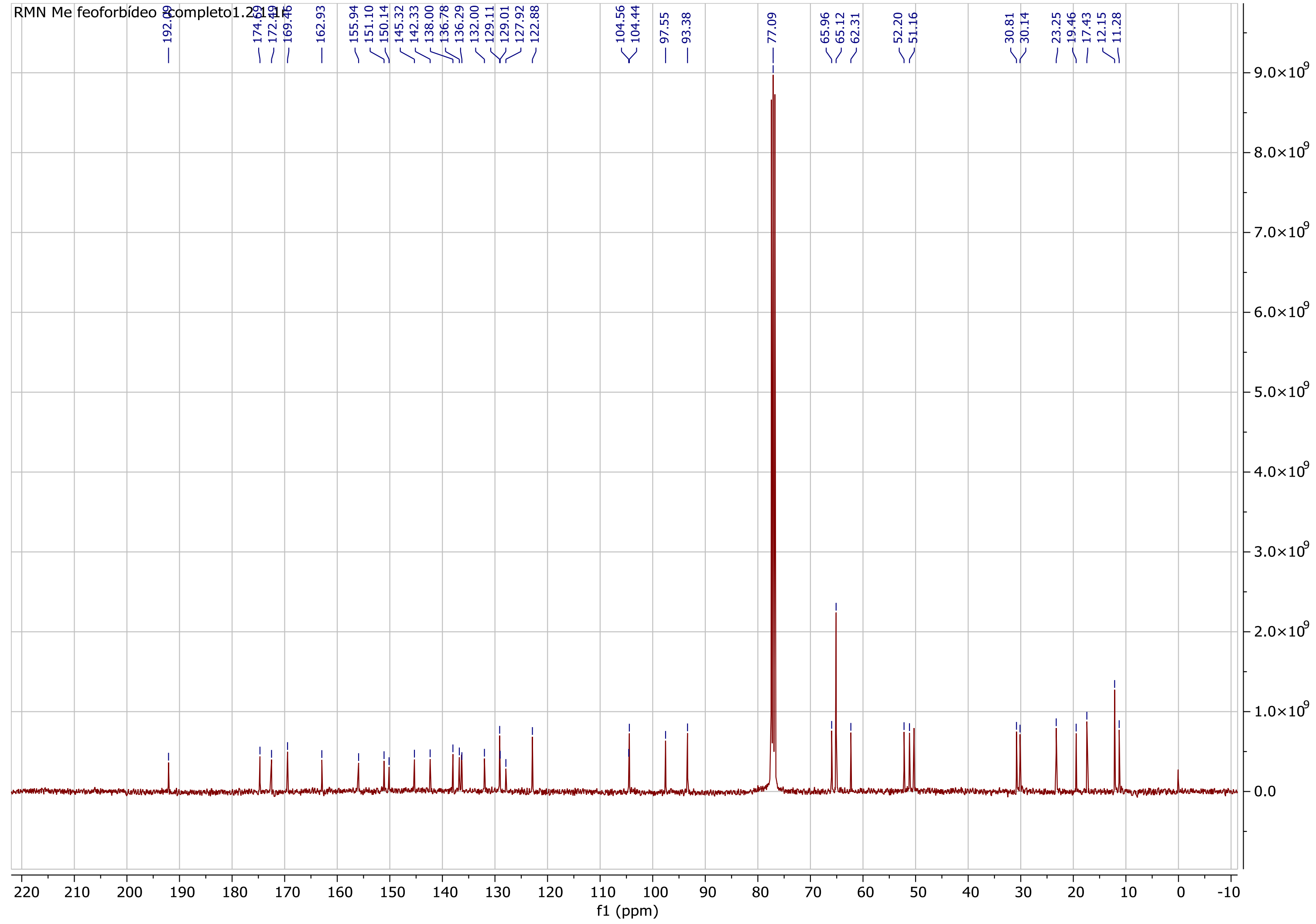

Figure S2: ^{13}C-NMR ($CDCl_3$) spectrum of methyl pheophorbide-*a* (**1**)

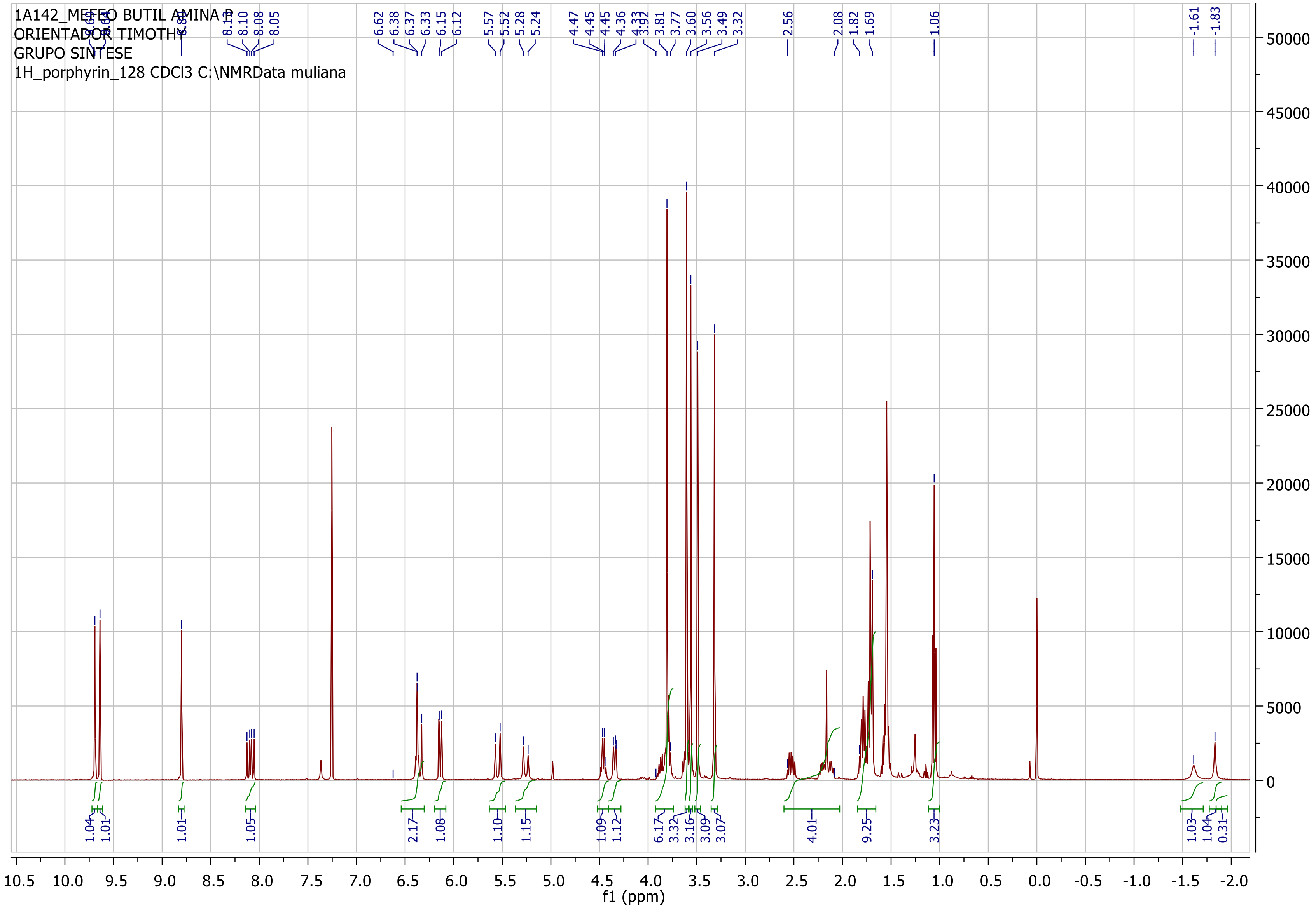

Figure S3: ^{1}H-NMR ($CDCl_3$) spectrum of 13-(Butylcarbamoyl)chlorin *e*6 15,17-dimethyl ester (**2**)

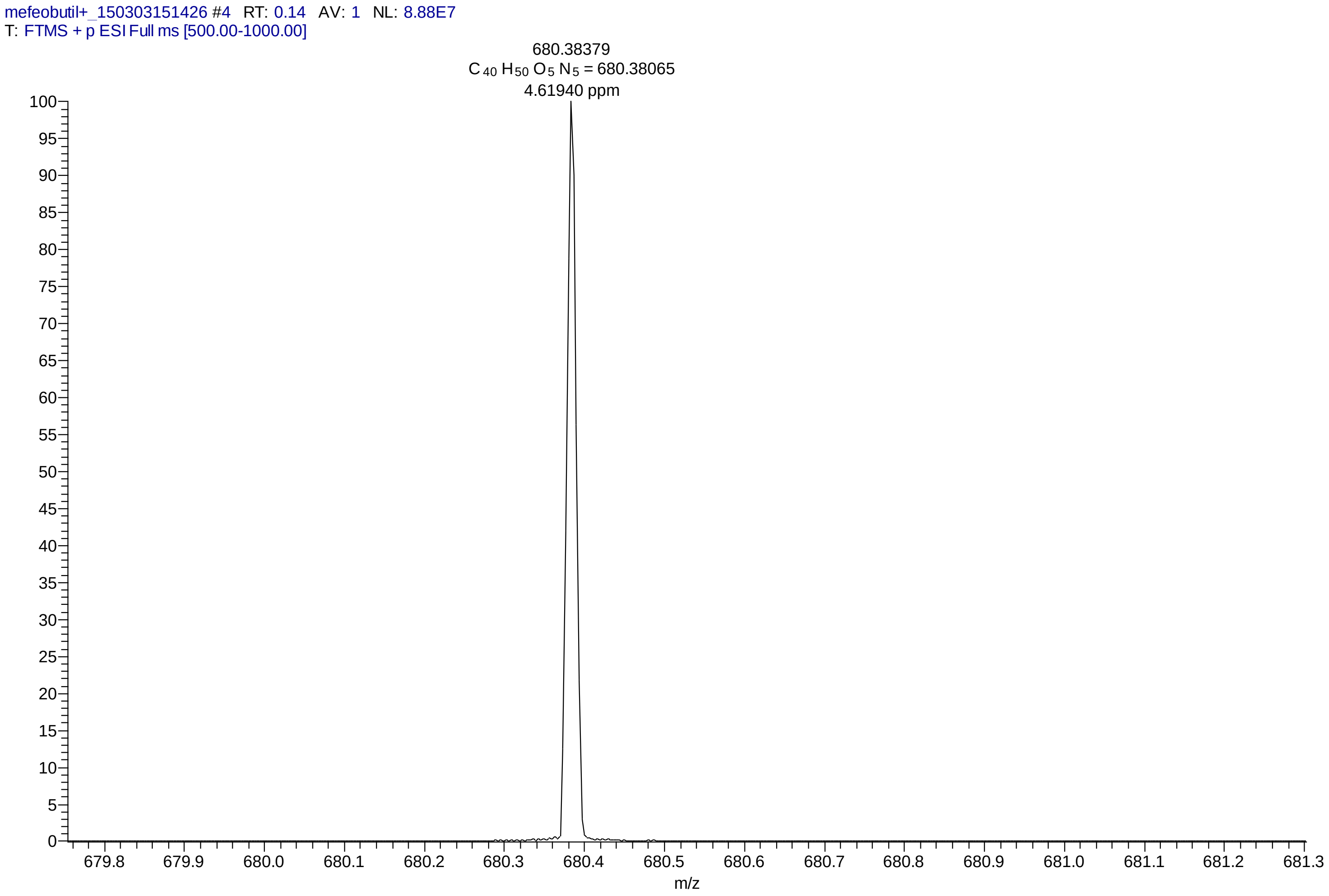

Figure S4: HRMS (ESI) of 13-(Butylcarbamoyl)chlorin *e*6 15,17-dimethyl ester (**2**)

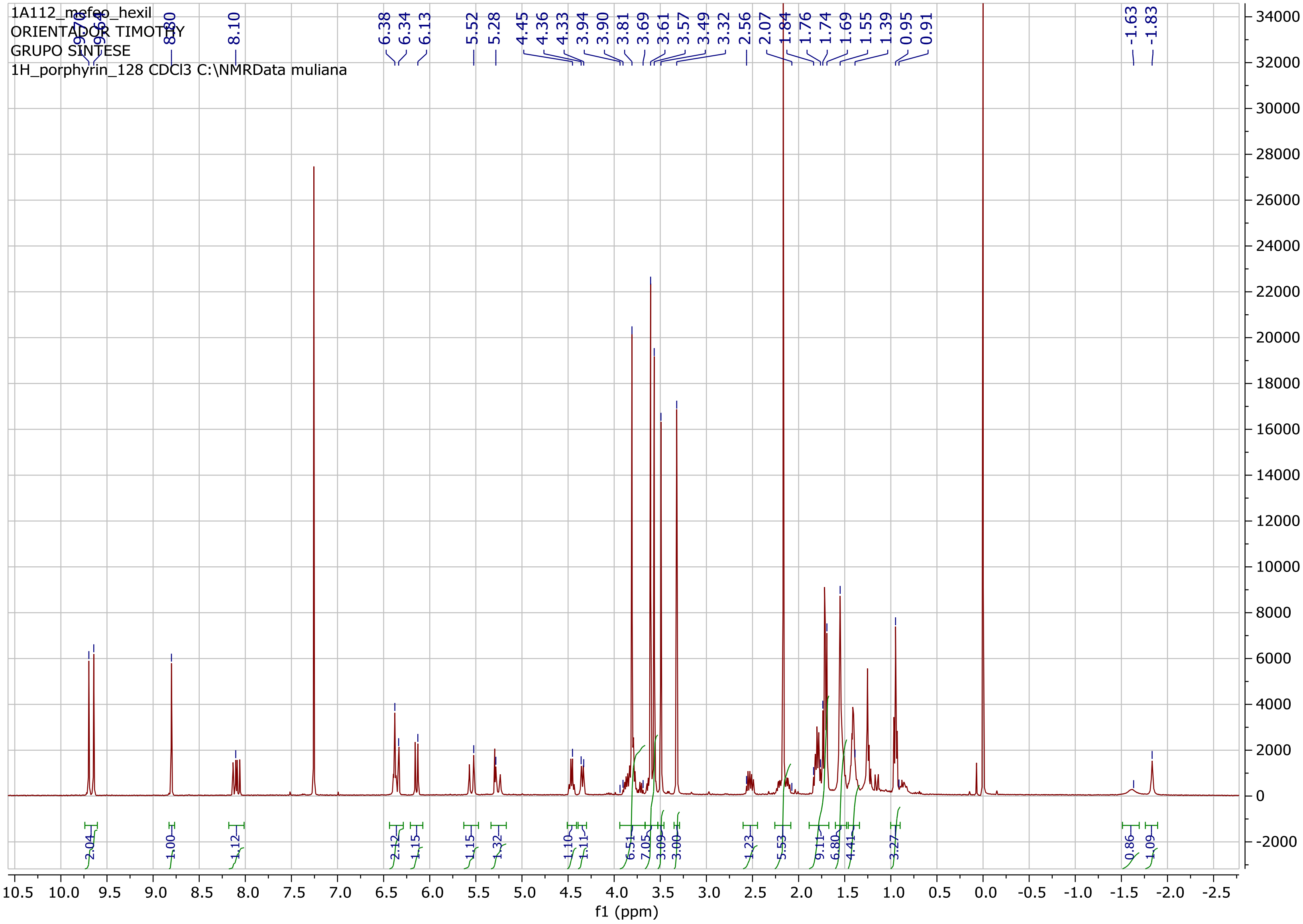

Figure S5: ^{1}H-NMR ($CDCl_3$) spectrum of 13-(Hexylcarbamoyl)chlorin *e*6 15,17-dimethyl ester (**3**)

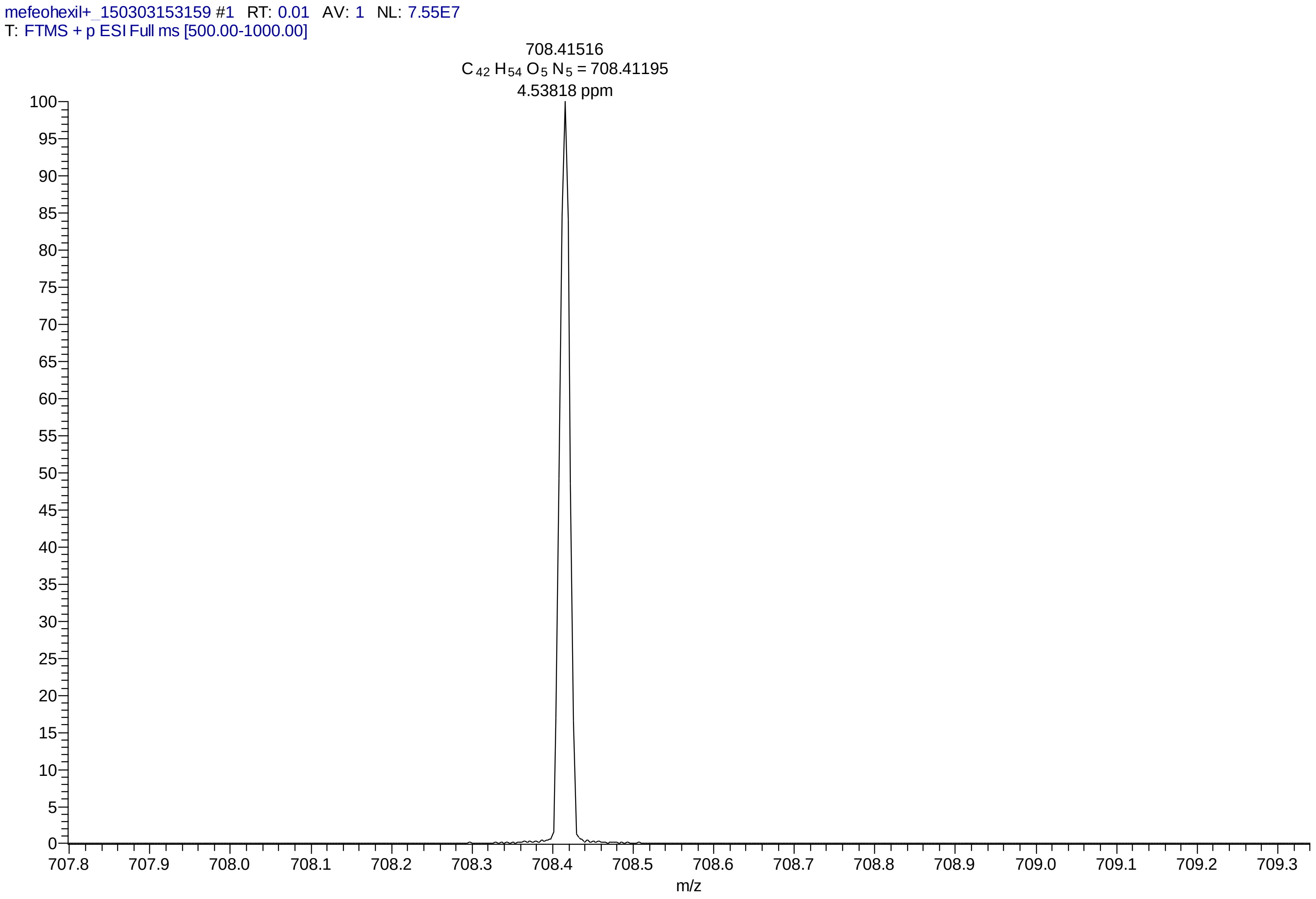

Figure S6: HRMS (ESI) of 13-(Hexylcarbamoyl)chlorin *e*6 15,17-dimethyl ester (**3**)

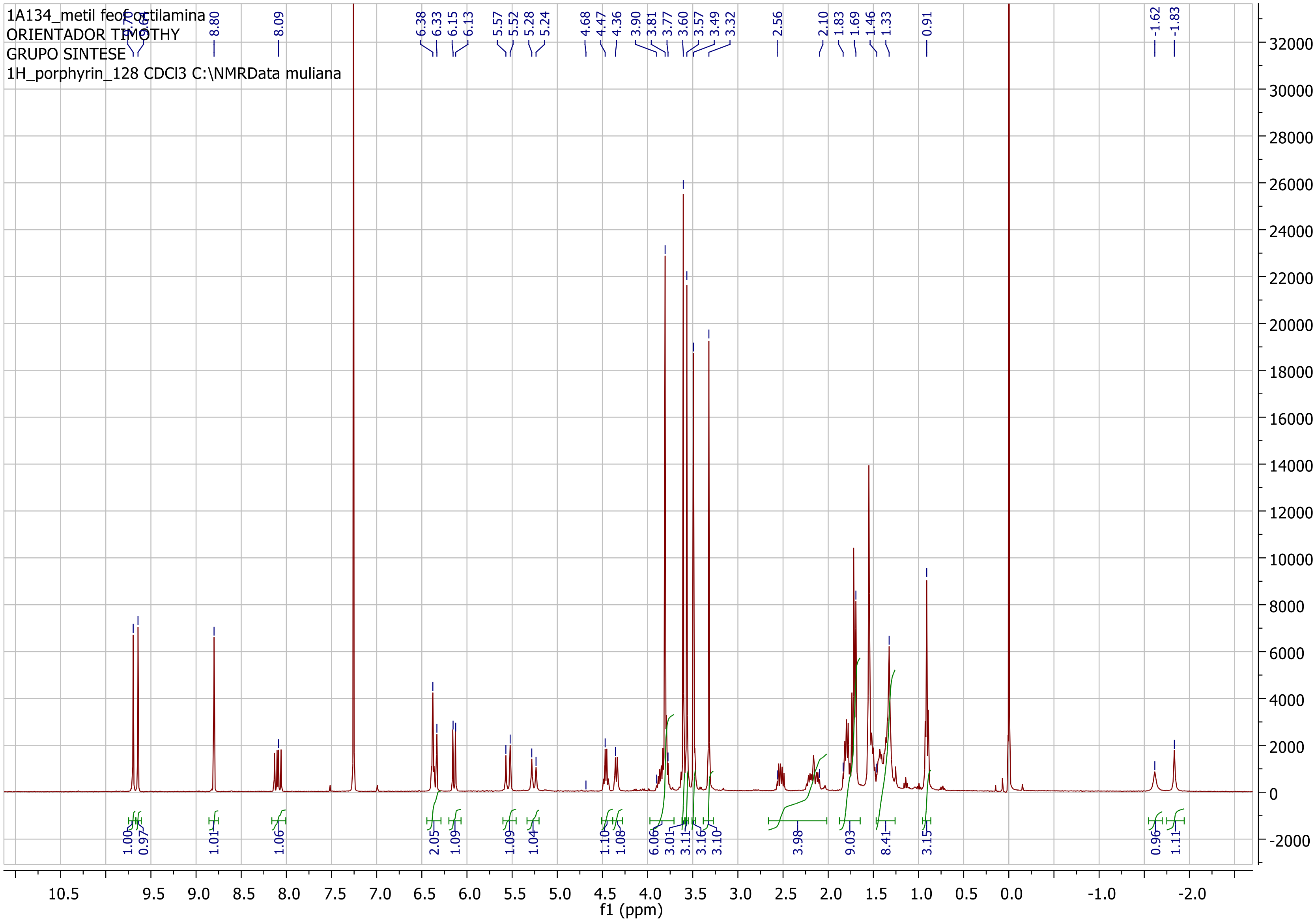

Figure S7: ^{1}H-NMR ($CDCl_3$) spectrum of 13-(Octylcarbamoyl)chlorin *e*6 15,17-dimethyl ester (**4**)

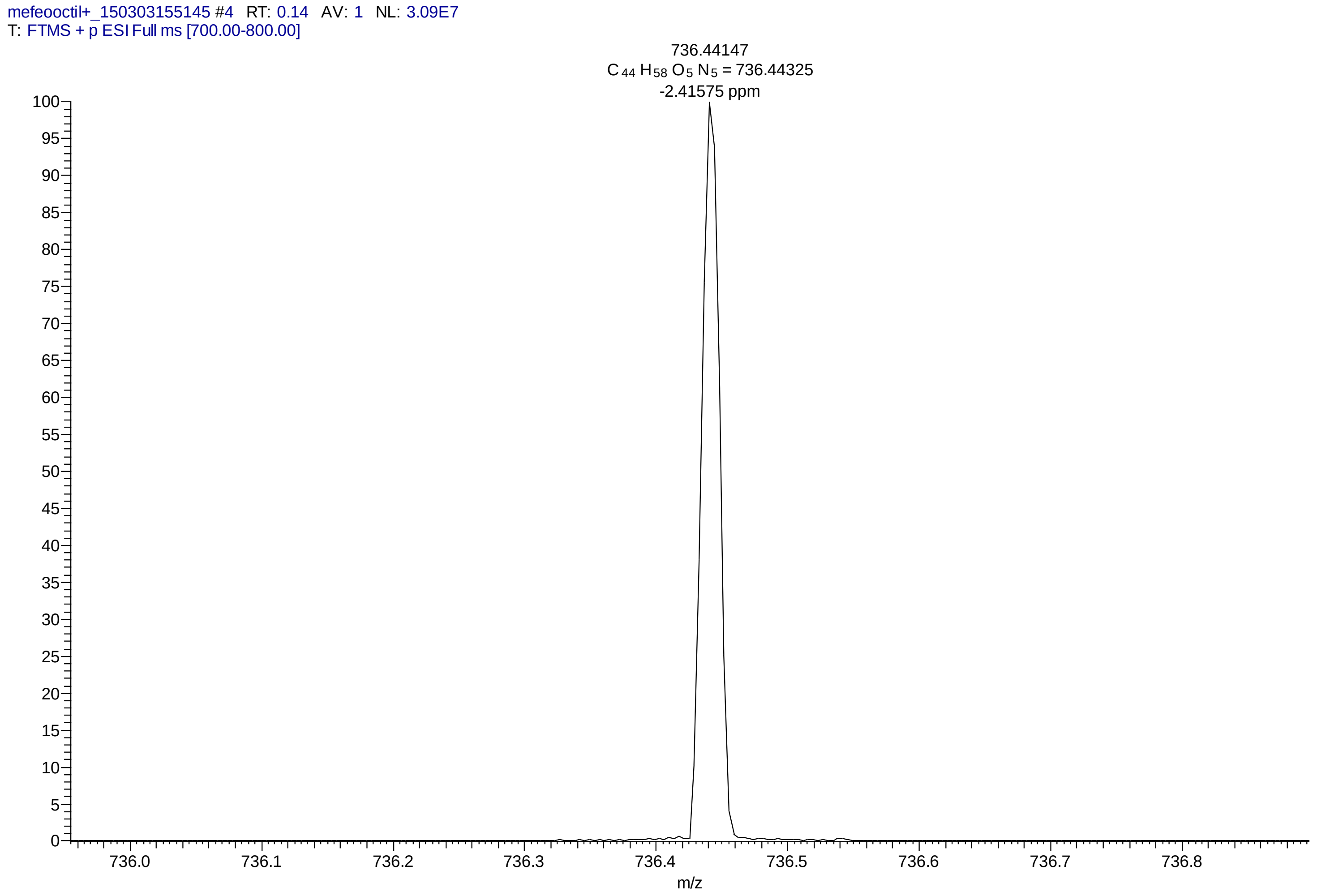

Figure S8: HRMS (ESI) of 13-(Octylcarbamoyl)chlorin *e*6 15,17-dimethyl ester (**4**)

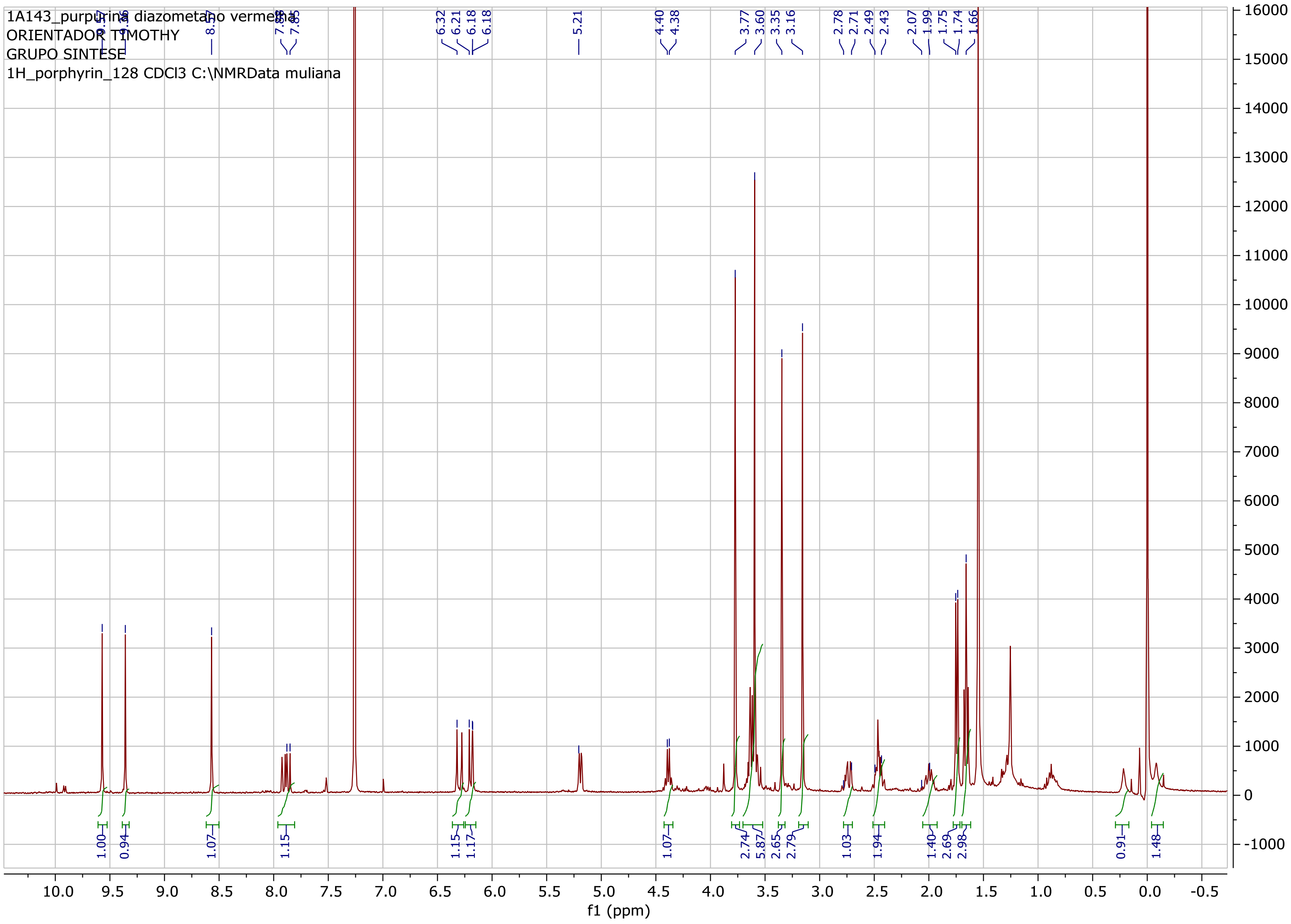

Figure S9: ^{1}H-NMR ($CDCl_3$) spectrum of Purpurin-18 Methyl Ester (**5**)

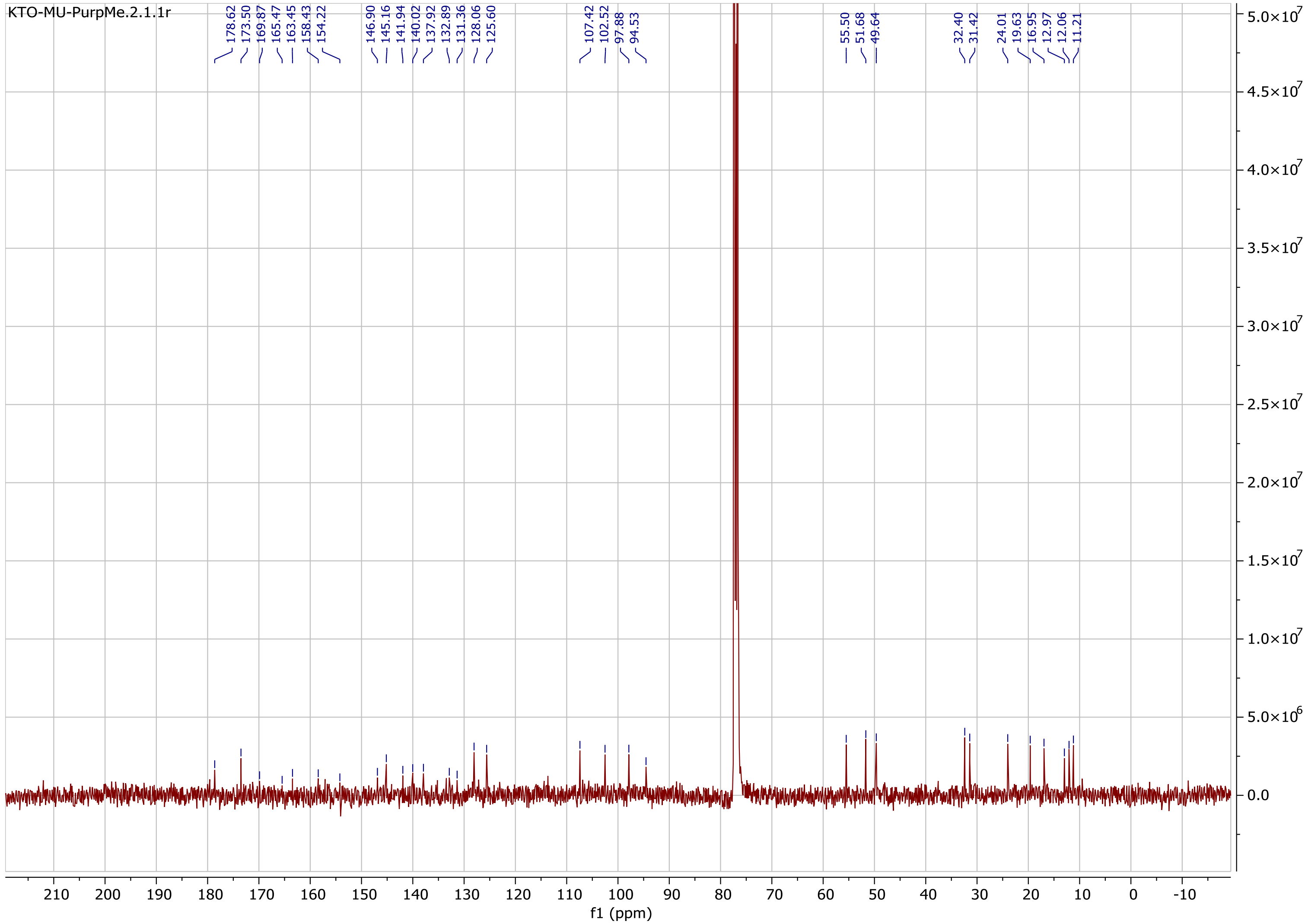

Figure S10: ^{13}C-NMR ($CDCl_3$) spectrum of Purpurin-18 Methyl Ester (**5**)

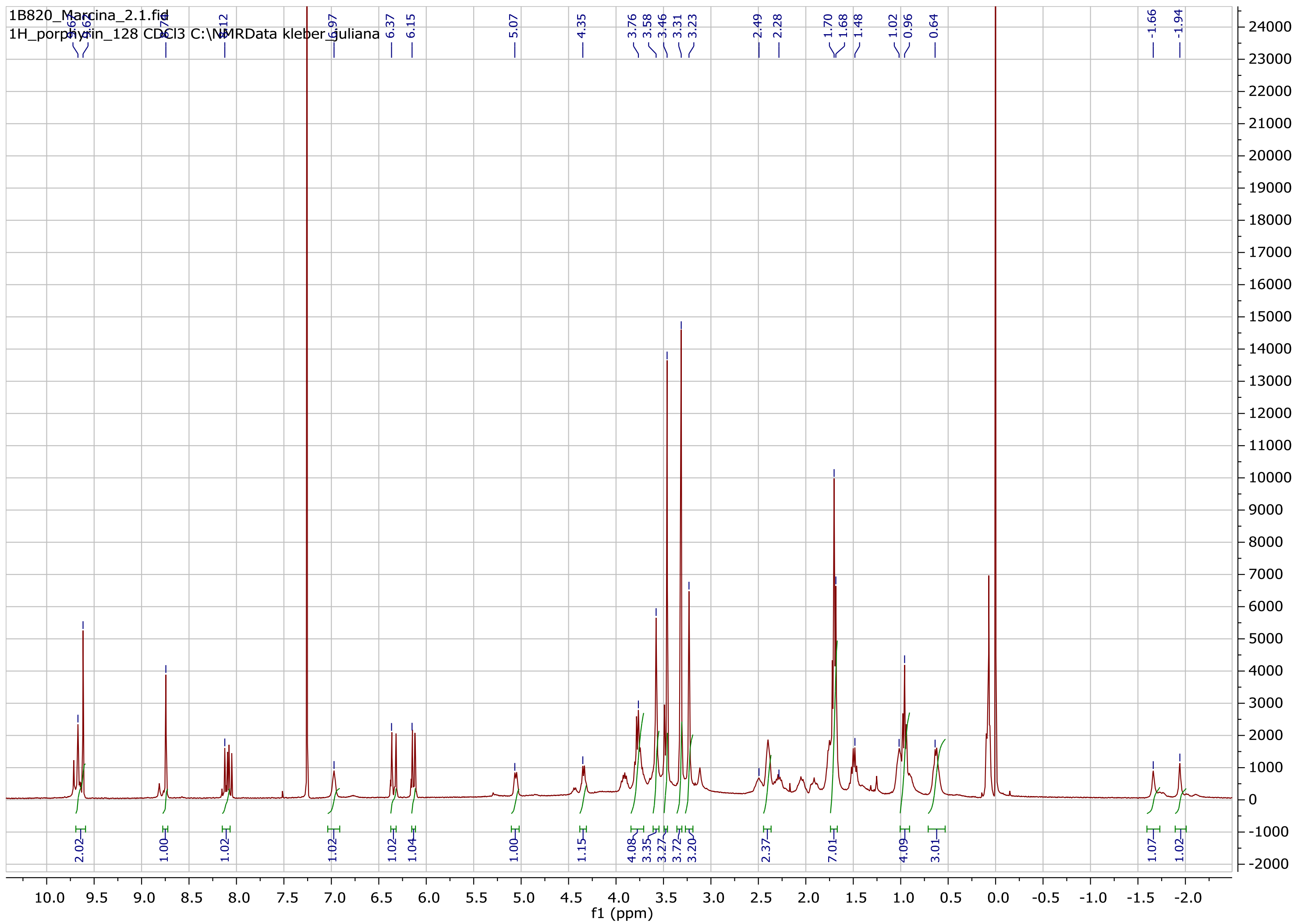

Figure S11: ^{1}H-NMR ($CDCl_3$) spectrum of Chlorin-p, 6-N-Butylamide-7-methyl Ester (**6**)

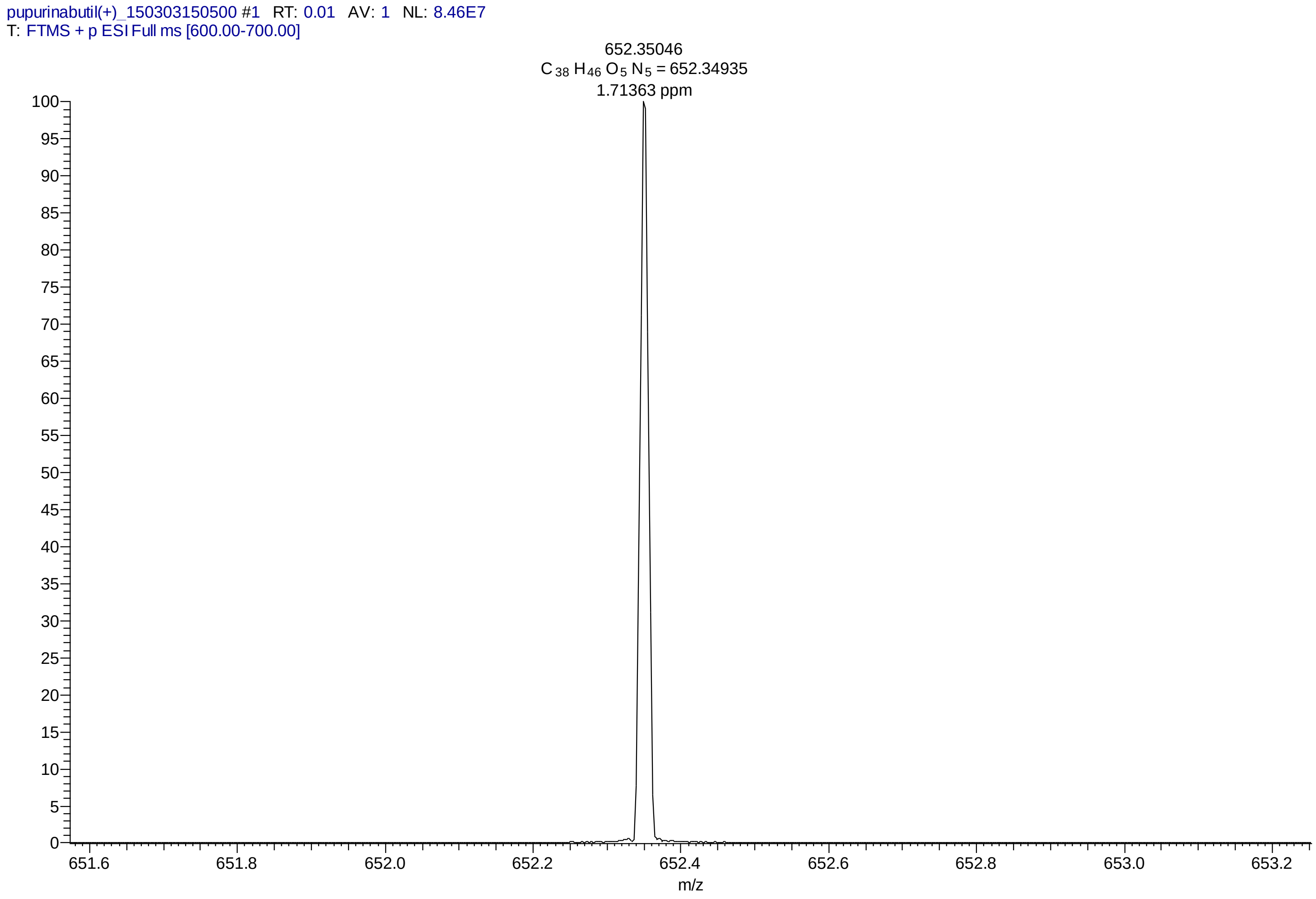

Figure S12: HRMS (ESI) of Chlorin-p, 6-N-Butylamide-7-methyl Ester (**6**)

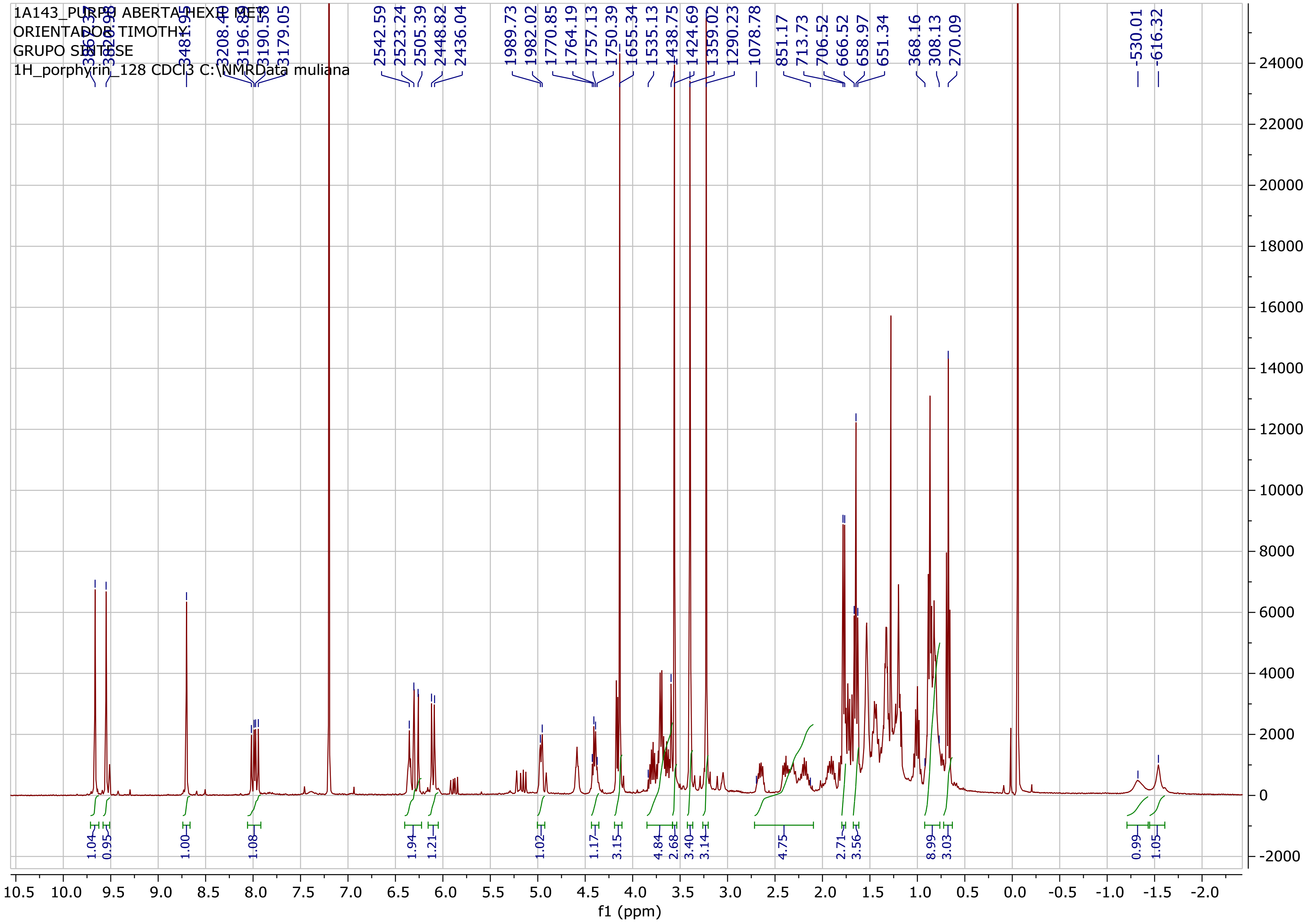

Figure S13: ^{1}H-NMR ($CDCl_3$) spectrum of Chlorin-p, 6-N-hexylamide-7-methyl Ester (**7**)

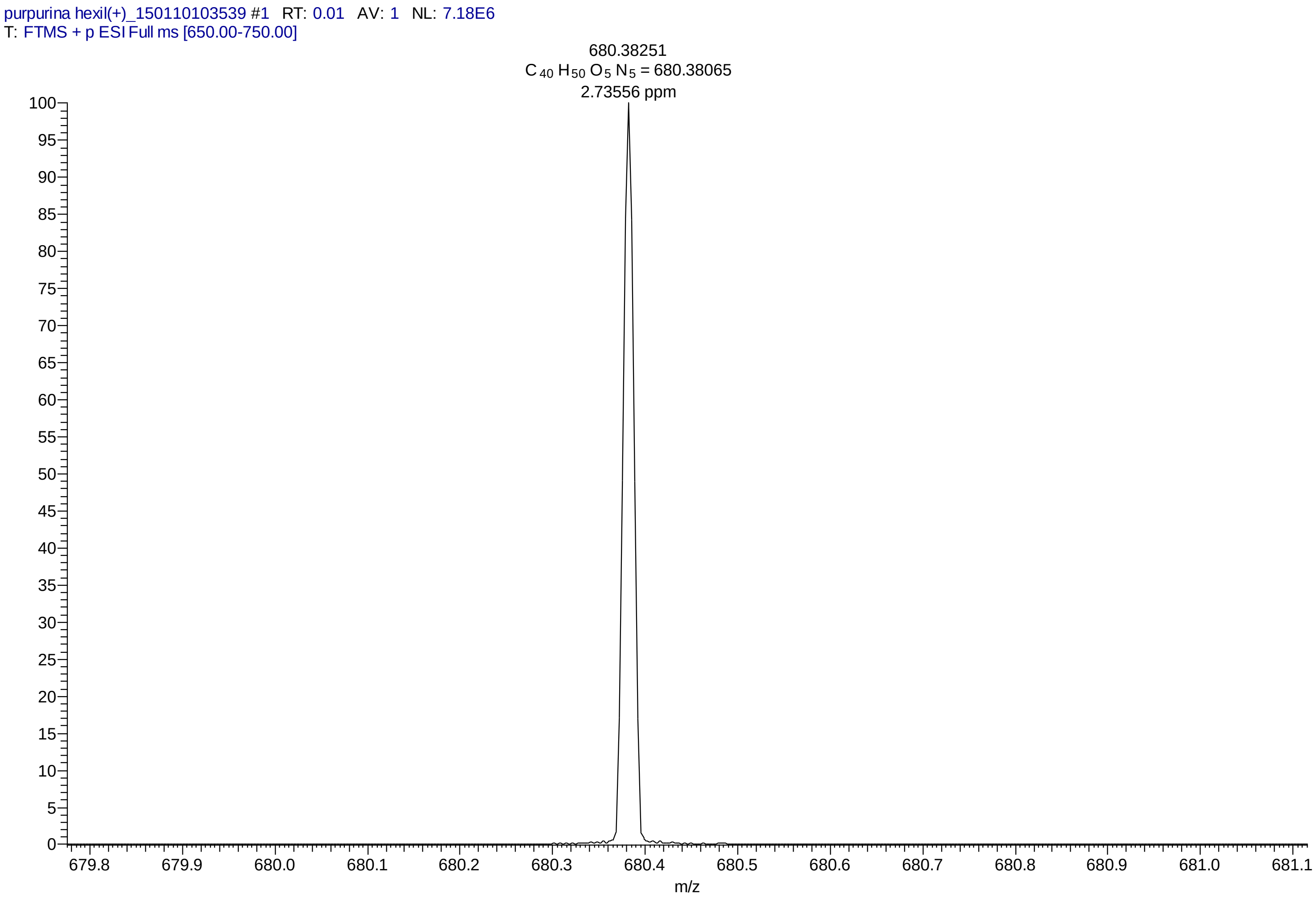

Figure S14: HRMS (ESI) of Chlorin-p, 6-N-hexylamide-7-methyl Ester (**7**)

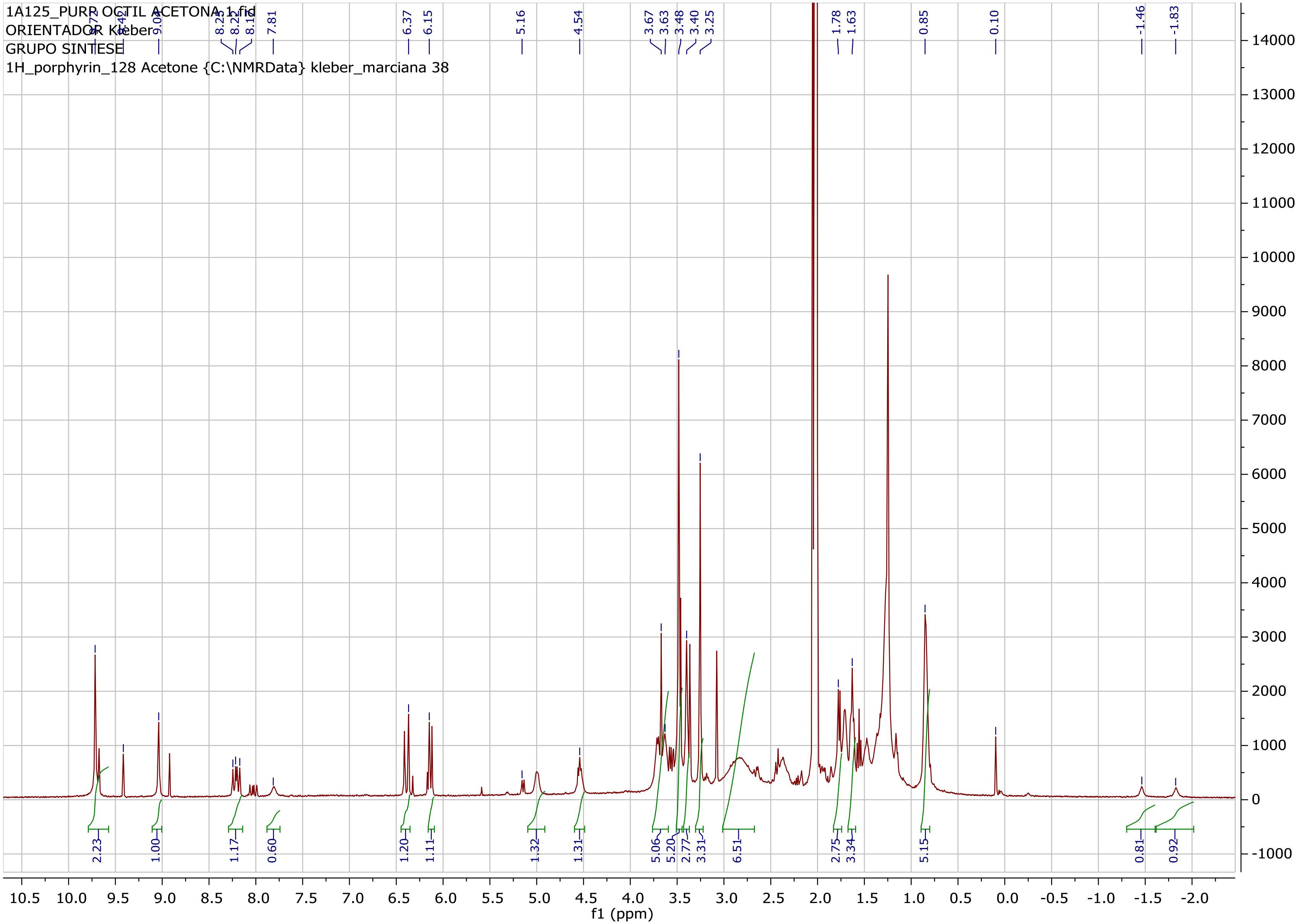

Figure S15: ^{1}H-NMR ($CDCl_3$) spectrum of Chlorin-p, 6-N-octylamide-7-methyl Ester (**8**)

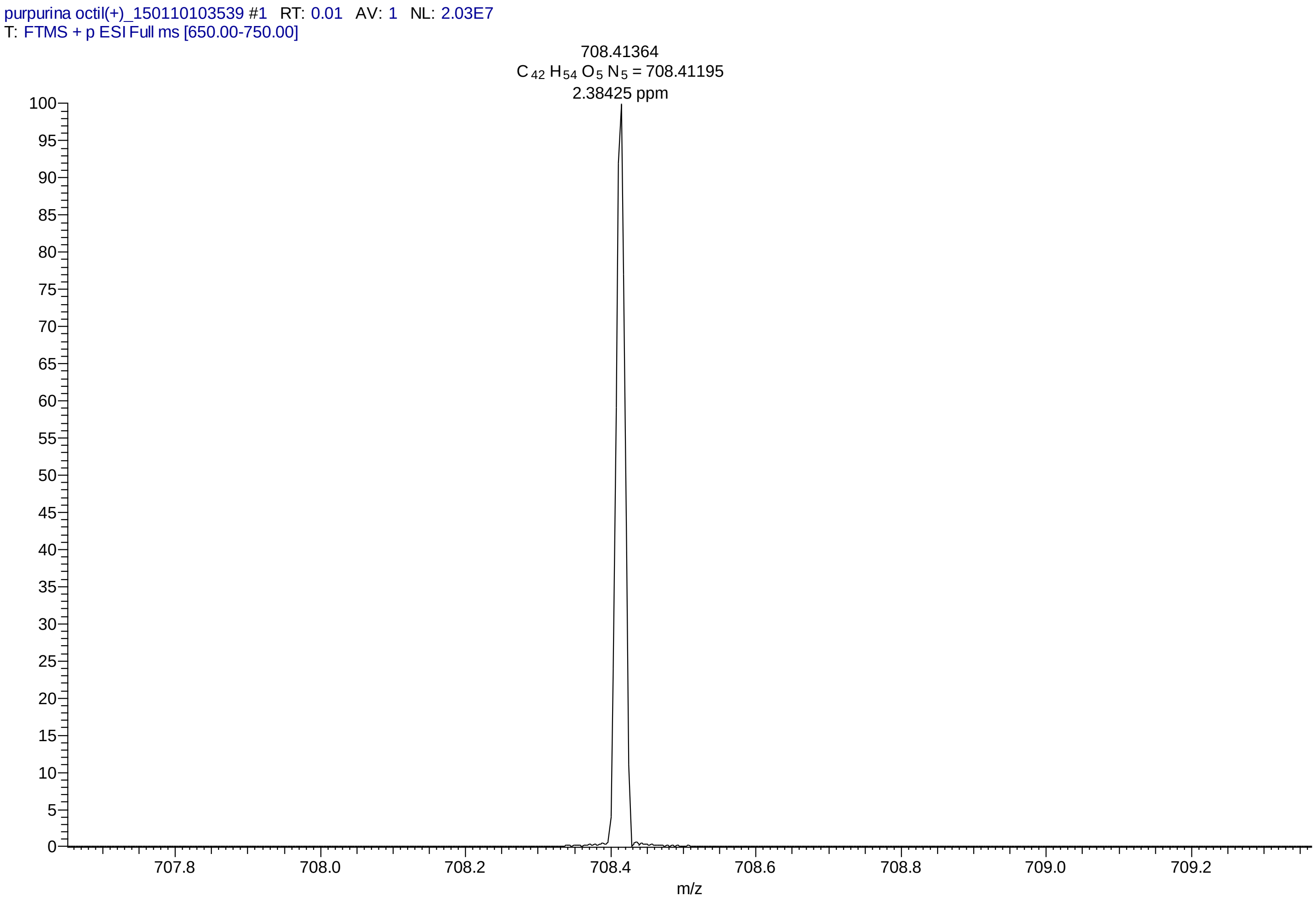

Figure S16: HRMS (ESI) of Chlorin-p, 6-N-octylamide-7-methyl Ester (**8**)

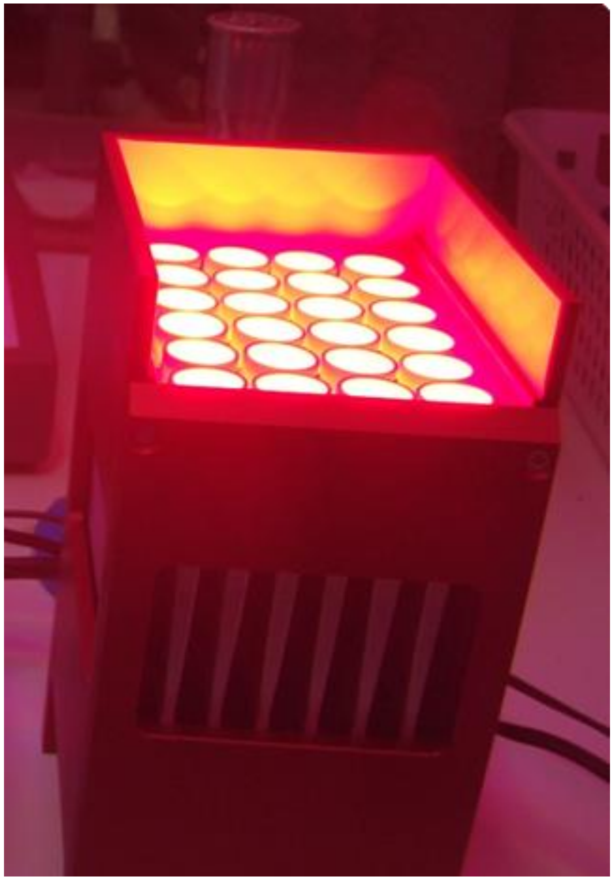

Figure S17: Homemade engineered Biotable model for PDI studies (660 nm).

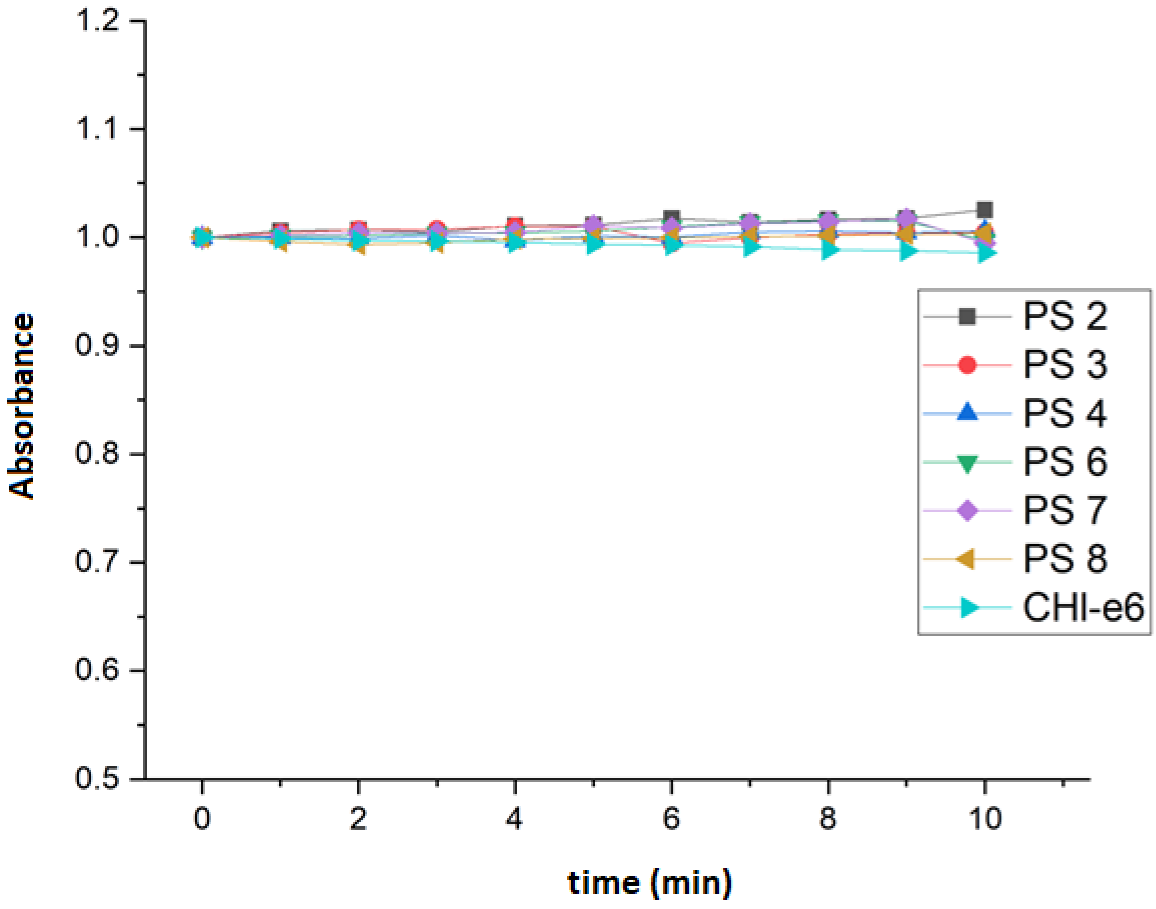

Figure S18: Photodegradation experiment at 660 nm at 63.7 mWcm-2 using ethyl acetate as solvent.

Table S1: Wavelengths data and Quantum yield of Singlet Oxygen $^1O_2$ data of the literature for some of the compounds.

PS	Our work (UV-Vis)	Literature data (UV-Vis) and Literature data Quantum yield of singlet oxygen $^1O_2$
Methyl pheophorbide-a (**1**)	λmax (CH_2Cl_2) (nm): 666, 609, 534, 505, 409.	λmax (CH_2Cl_2) 668 (44 600), 610 (8 620), 538 (9 710), 506 (10 800), 412 (106 000)[1]<br><br>Quantum yield of singlet oxygen $^1O_2$<br>0.44 (in benzene); 0.47 (in 1-octanol)[2]
13-(Butylcarbamoyl)-chlorin e6 15,17-dimethyl ester (**2**)	λmax (CH_2Cl_2) (nm): 662, 607, 528, 498, 399.	λ max, nm ($CHCl_3$) 664, 608, 558, 529, 501 402.[3]
13-(Hexylcarbamoyl)chlorin e6 15,17-dimethyl ester (**3**)	*λmax* (CH_2Cl_2) (nm): 663, 605, 527, 497, 398.	*λ, nm* ($CHCl_3$): 664, 609, 558, 528, 501, 402.[3]
13-(Octylcarbamoyl)chlorin e6 15,17-dimethyl ester (**4**)	*λmax* (CH_2Cl_2) (nm): 663, 609, 527, 497, 399.	UV-vis ($CHCl_3$): λ, nm (log ε) 664, 607, 558, 530, 502, 402. [3]
Purpurin-18 Methyl Ester (**5**)	*λmax (CH_2Cl_2) (nm):* 699, 642, 546, 508, 478, 410.	λmax (nm) ($CHCl_3$): ($\varepsilon.10^{-3}$): 360 (22.9); 413 (44.6); 481 (1.9); 510 (3.9); 548 (10.9);646 (4.7); 701 (23.2)[4]<br><br>Quantum yield of singlet oxygen $^1O_2$<br>0.55 (in ethanol); 0.73 (in toluene); 0.80 (in pyridine)[5]
Chlorin-p, 6-N-Butylamide-7-methyl Ester (**6**)	*λmax* (CH_2Cl_2) (nm): 662, 606, 526, 498, 398.	*λmax/nm* 664 (ε 4.56 x 10^4), 608 (1.08 x 10^4), 532(1.13 x 10^4), 500(1.90 x 10^4) and 404(1.37 x 10^5)[6]

Chlorin-p, 6-N-hexylamide-7-methyl Ester (**7**)	*λmax* (CH_2Cl_2) (nm): 666, 608, 528, 497, 398.	*λmax* THF/CH_2Cl_2 (1:4) (nm): 666 (1.78 x 10^4), 612 (1.85 x 10^3), 528 (1.73 x 10^3), 498 (4.92 x 10^3), 402 (5.11 x 10^4)[7]
Chlorin-p, 6-N-octylamide-7-methyl Ester (**8**)	*λmax* (CH_2Cl_2) (nm): 663, 605, 528, 499, 399.	Don't have this date

Reference:

[1] Ma, L; Dolphin, D.; Nucleophilic reaction of 1,8- diazabicyclo[5.4.0]undec-7-ene and 1,5- diazabicyclo[4.3.0]non-5-ene with methyl pheophorbide a. Unexpected products. *Tetrahedron*, **1996**, *52* (*3*), 849-860. https://doi.org/10.1016/0040-4020(95)00944-2.

[2] Kustov, A, V.; Belykh, D.V.; Startseva, O. M.; Kruchin, S. O.; Venediktov, E. A.; Berezin, D. B. Pharm Anal Acta. **2016**, *7(5*), 1-5. https://doi.org/10.4172/2153-2435.1000480.

[3] Belykh, D. V.; Tarabukinaa, I. S.; Gruzdevb, I. V.; Kodessc, M. I.; Kutchin, A. V. Aminomethylation of chlorophyll a derivatives using bis(N,N-dimethylamino)methane. Porphyrins Phthalocyanines **2009**; *13*, 949–956. https://doi.org/10.1142/S1088424609001133.

[4] Drogat, N.; Barriere, M.; Granet, R.; Sol, V.; Krausz, P. High yield preparation of purpurin-18 from Spirulina maxima. *Dyes Pigm*. **2011**, *88 (1)*, 125-127, https://doi.org/10.1016/j.dyepig.2010.05.006.

[5] Redmond, R. W.; Gamlin, J. N. A Compilation of Singlet Oxigen Yields from Biologically Relevant Molecules. *Photochem. Photobiol*. **1999**, *70 (4)*, 391- 475. https://doi.org/10.1111/j.1751- 1097.1999.tb08240.x

[6] Lee, S. J. H.; Jagerovic, N.; Smith, K. M. Use of the Chlorophyll derivative, Purpurin-18, for Syntheses of Sensitizers for use in Photodynamic Therapy. *Journal of the Chemical Society-Perkin Transactions 1*. ***1993***, *(19), 2369-2377*. https://doi.org/*10.1039/p19930002369*

[7] Journal of Medicinal Chemistry (2001), 44(10), 1540-1559. https://scifinder-n-cas.ez350.periodicos.capes.gov.br/searchDetail/reaction/62fbd8f0fd474116c001ae34/reactionDetails CAS Method Number 3-366-CAS-9701836
